# A digital health algorithm to guide antibiotic prescription in pediatric outpatient care: a cluster randomized controlled trial

**DOI:** 10.1038/s41591-023-02633-9

**Published:** 2023-12-18

**Authors:** Rainer Tan, Godfrey Kavishe, Lameck B. Luwanda, Alexandra V. Kulinkina, Sabine Renggli, Chacha Mangu, Geofrey Ashery, Margaret Jorram, Ibrahim Evans Mtebene, Peter Agrea, Humphrey Mhagama, Alan Vonlanthen, Vincent Faivre, Julien Thabard, Gillian Levine, Marie-Annick Le Pogam, Kristina Keitel, Patrick Taffé, Nyanda Ntinginya, Honorati Masanja, Valérie D’Acremont

**Affiliations:** 1 Centre for Primary Care and Public Health (Unisanté), University of Lausanne, Lausanne, Switzerland; 2 Ifakara Health Institute, Dar es Salaam, United Republic of Tanzania; 3 Swiss Tropical and Public Health Institute, Allschwil, Switzerland; 4 University of Basel, Basel, Switzerland; 5 National Institute of Medical Research – Mbeya Medical Research Centre, Mbeya, United Republic of Tanzania; 6 Pediatric Emergency Department, Department of Pediatrics, University Hospital Bern, Bern, Switzerland

**Keywords:** Paediatrics, Outcomes research, Infectious diseases

## Abstract

Excessive antibiotic use and antimicrobial resistance are major global public health threats. We developed ePOCT+, a digital clinical decision support algorithm in combination with C-reactive protein test, hemoglobin test, pulse oximeter and mentorship, to guide health-care providers in managing acutely sick children under 15 years old. To evaluate the impact of ePOCT+ compared to usual care, we conducted a cluster randomized controlled trial in Tanzanian primary care facilities. Over 11 months, 23,593 consultations were included from 20 ePOCT+ health facilities and 20,713 from 20 usual care facilities. The use of ePOCT+ in intervention facilities resulted in a reduction in the coprimary outcome of antibiotic prescription compared to usual care (23.2% versus 70.1%, adjusted difference −46.4%, 95% confidence interval (CI) −57.6 to −35.2). The coprimary outcome of day 7 clinical failure was noninferior in ePOCT+ facilities compared to usual care facilities (adjusted relative risk 0.97, 95% CI 0.85 to 1.10). There was no difference in the secondary safety outcomes of death and nonreferred secondary hospitalizations by day 7. Using ePOCT+ could help address the urgent problem of antimicrobial resistance by safely reducing antibiotic prescribing. Clinicaltrials.gov Identifier: NCT05144763

## Abstract (web summary)

A cluster randomized trial in Tanzania showed that the implementation of a decision support algorithm decreased antibiotic prescriptions to children considerably, without impacting clinical outcomes.

## Main

Bacterial antimicrobial resistance (AMR) was responsible for 1.27 million deaths in 2019, with the highest burden in sub-Saharan Africa^1^. This is as many deaths as malaria and human immunodeficiency virus (HIV) combined. Inappropriate and excessive prescription of antibiotics represents one of the primary contributors to AMR^2–4^. In Tanzania and many resource-constrained countries, more than 50% of sick children receive antibiotics when visiting a health facility^5–8^, with 80–90% of such antibiotics prescribed at the outpatient level^6,9,10^ and most deemed inappropriate^5,9–11^. Antibiotic use and AMR are projected to increase over the coming years, indicating the urgency to take action^12–14^. Accordingly, the World Health Organization has declared AMR as “one of the biggest threats to global health, food security and development today”^15^. In response, countries worldwide, including Tanzania, have developed national action plans on antimicrobial resistance to address this important problem^16,17^.

Electronic clinical decision support algorithms (CDSAs) are digital health or mobile health tools that guide health-care providers on what symptoms and signs to assess, advise on what tests to perform, and propose the appropriate diagnoses, treatments and management^18,19^. Previous efficacy studies under controlled research conditions have shown the potential for digital CDSAs to reduce antibiotic prescription in children 2 to 59 months old^20,21^. However, many close-to-real-world studies have shown little to no reduction in antibiotic prescription^22–24^. In addition, many of the close-to-real-world studies have a number of methodological limitations as health facilities were not randomized and/or safety was not evaluated^18,24,25^, emphasizing the need for more evidence on the impact of CDSAs on antibiotic prescription. Finally, poor uptake remains a challenge with previous and existing CDSAs^26,27^.

We developed ePOCT+, a new CDSA with point-of-care tests, to address these challenges^28^. The scope of ePOCT+ was expanded from previous versions of the CDSA^20,29^ to include infants under 2 months and children up to age 14 years, and to address syndromes and diagnoses not considered by other CDSAs^30^. The aim of this study was to evaluate the impact of ePOCT+ compared to usual care on antibiotic prescription and day 7 clinical outcome in a pragmatic, cluster randomized controlled trial in acutely sick children under 15 years of age presenting to Tanzanian primary care facilities.

## Result

### Baseline characteristics of health facilities and patients

**Fig. 1:**
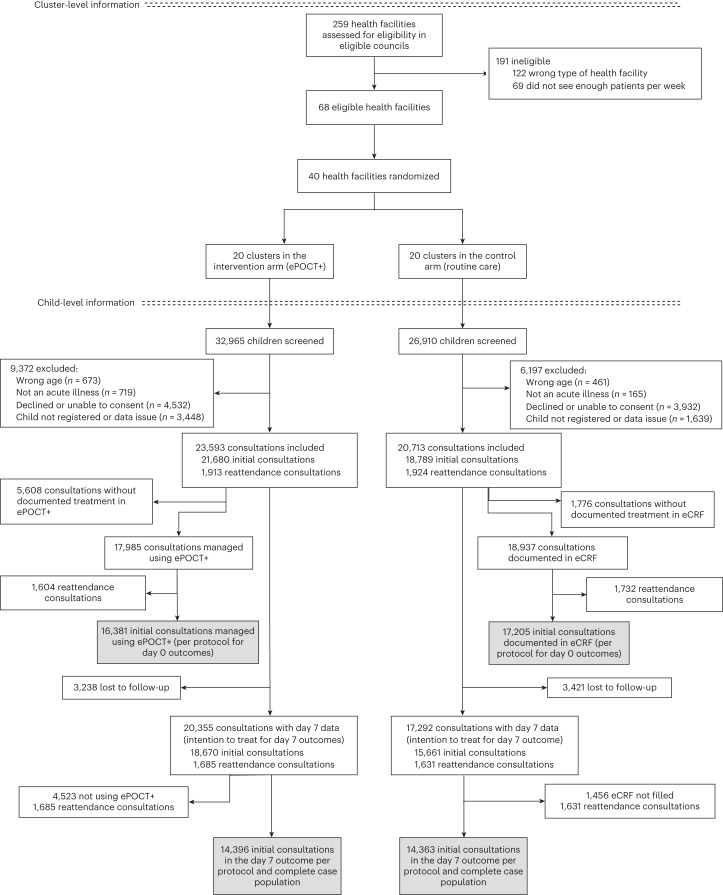
Health facility and patient flow diagram. Boxes highlighted in gray correspond to the coprimary outcome populations.

A total of 68 out of 259 health facilities from the participating councils met the eligibility criteria (Fig. 1). One hundred twenty-two health facilities were ineligible as they were either hospitals or private dispensary or health centers, and 69 did not see enough patients per week. A stratified random sampling process identified 40 health facilities for inclusion in the study (24 in the Morogoro region and 16 in the Mbeya region), which were randomized 1:1 to ePOCT+ (intervention) or usual care (control). Overall, 59,875 children were screened for inclusion between 1 December 2021 and 31 October 2022, and 44,306 (74%) consultations were enrolled (23,593 in ePOCT+ health facilities and 20,713 in usual care health facilities). The first health facilities started enrolling patients on 1 December 2021, and the last health facilities started enrolling patients on 13 April 2022. A total of 28,243 unique patients were enrolled with a mean of 1.6 consultations per patient over the duration of the study. Among those enrolled in the intervention health facilities, 17,985 (76.2%) consultations were managed using ePOCT+, and day 7 outcome was ascertained in 20,355 consultations (86.3%). In usual care health facilities, 18,937 (91.4%) consultations had final treatment documented in the electronic case report form (eCRF), and 17,292 consultations (83.5%) had day 7 outcome ascertained. Information technology (IT) problems and power outages were reported by research assistants on respectively 293 (7.3%) and 245 (6.1%) health facility days in ePOCT+ facilities, and 160 (4.1%) and 245 (6.1%) health facility days in usual care facilities. Both issues contributed to children being prevented from enrollment in the study.

Intervention health facilities saw similar numbers of consultations, but had a slightly lower Service Availability and Readiness Assessment Pediatric score (Table 1)^31^. Patients in both study arms were similar in age, sex, type of consultation and previous hospitalization (Table 1). Malaria prevalence among those tested was similar in both study arms (Table 1). Young infants less than 2 months of age in the intervention health facilities presented more frequently for fever, convulsions or lethargy, and slightly less often for respiratory conditions, whereas patients 2 months and above had a similar distribution in presenting complaints (Table 2). Age, phone availability and level of health facility differed among patients with and without day 7 outcome ascertained (Supplementary Tables 1 and 2). Patients managed and not managed per protocol were similar, except for the level of health facility (Supplementary Table 3).

**Table 1:** Baseline characteristics of enrolled participants and health facilities

Health facilities	ePOCT+ (*n* = 20)	Usual care (*n* = 20)
Level of health facility, *n*		
Dispensaries	16	16
Health centers	4	4
Region, *n*		
Morogoro	12	12
Mbeya	8	8
Number of enrolled patients per health facility per month, median (IQR)	127 (101; 199)	136 (73; 163)
Service availability and readiness assessment^a^		
General Service Readiness score, % (mean ± s.d.)	60.3 ± 10.8	63.7 ± 9.4
Pediatric score, % (mean ± s.d.)	55.9 ± 10.8	64.9 ± 10.6

Participants	ePOCT+ (*n* = 23,593)	Usual care (*n* = 20,713)
Sex: Female, % (*n*)	51.2 (12,085)	51.3 (10,075)
Age, days, median (IQR)	583 (263; 1,202)	555 (246; 1,189)
Age group, % (*n*)		
0 to <2 months	4.0 (954)	5.0 (1,038)
2 to <60 months	84.1 (19,845)	82.0 (16,984)
5 to <15 years	11.8 (2,794)	13.0 (2,691)
Type of consultation, % (*n*)		
New consultation	91.9 (21,680)	90.7 (18,789)
Reattendance	7.8 (1,841)	9.2 (1,899)
Referral from another health facility	0.3 (72)	0.1 (25)
Positive malaria test among those tested, % (*n*/*N*)	18.4 (1,878/10,225)	19.2 (1,803/9,378)
Hospitalized in the last 14 days, % (*n*)	0.3 (65)	0.4 (73)
Phone number available, % (*n*)	84.0 (19,808)	83.0 (17,186)

Participant data from all enrolled patients. Values of standard deviations (s.d., after mean values) are preceded by the ± sign. IQR, interquartile ranges (after median values).

^a^Scores were calculated based on the proportion of prespecified indicators that were present in each health facility during the assessment of health facilities before the start of the study^31^.

**Table 2:** Presenting complaints of infants and children under 15 years old

Presenting complaints, %	ePOCT+	Usual care
Infants <2 months	*n* = 717	*n* = 929
Fever, convulsions, lethargy	25.7	13.1
Respiratory	43.7	46.8
Gastrointestinal	22.2	19.7
Skin	14.0	14.5
Ear/mouth	2.5	0.9
Eye	7.0	5.4
Feeding/weight	0.3	1
Malformation	0.4	0.4
Injuries	0.4	0.1
Other	6.4	7.8
Infants and children ≥2 months to <15 years	*n* = 17,268	*n* = 17,089
Fever	61.6	56.9
Respiratory (cough/difficulty breathing)	47.8	49.4
Gastrointestinal (diarrhea/vomiting)	23.4	22.3
Skin	12.5	11.9
Ear/throat/mouth	2.6	2.3
Eye	2.1	2.1
Genitourinary	1.4	3.1
Neurological (headache, stiff neck)	3	1.2
Accident/musculoskeletal (including burns, wounds, poison)	1.5	2.0
Other	2.1	4.2

Data from patients for whom clinical information was entered into ePOCT+ in the intervention arm, and in the eCRF in control health facilities (per protocol population). Patients may have multiple complaints.

### Primary outcomes: antibiotic prescription and clinical failure

**Fig. 2:**
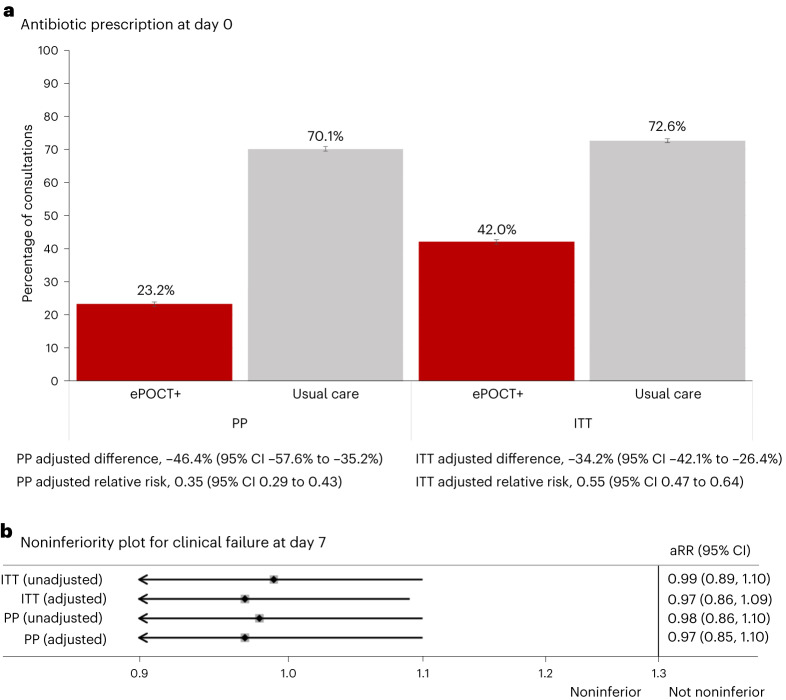
Coprimary outcomes. **a**, Proportion of antibiotic prescription in ePOCT+ and usual care health facilities; data are presented as the point estimate and unadjusted 95% confidence intervals. Sample sizes are as follows: PP ePOCT+ clusters *n* = 16,381, PP usual care clusters *n* = 17,205, ITT ePOCT+ clusters *n* = 21,680, ITT usual care clusters *n* = 18,789. **b**, Relative risk of day 7 clinical failure between ePOCT+ and usual care health facilities, with noninferiority prespecified as an adjusted relative risk of <1.3. Noninferiority plot shown on a logarithmic scale. ITT, intention to treat; PP, per protocol; aRR, adjusted relative risk.

Overall antibiotic prescription at initial consultations for the per protocol analysis was 23.2% (3,806 of 16,381) in ePOCT+ health facilities and 70.1% (12,058 of 17,205) in routine care health facilities, which corresponds to an adjusted absolute difference of −46.4% (95% CI −57.6 to −35.2) (Table 3 and Fig. 2). The adjusted analysis found a 65% reduction in the risk of prescribing an antibiotic at day 0 (adjusted relative risk (aRR) 0.35, 95% CI 0.29 to 0.43, *P* < 0.001). Using a conservative imputation analysis approach in the intention-to-treat population by considering that all patients who were not managed per protocol were prescribed an antibiotic, antibiotic prescription remained lower in ePOCT+ health facilities than in usual care, with an adjusted absolute difference of −34.2% (95% CI −42.1% to −26.4%) (Extended Data Table 1). When including reattendance cases, antibiotic prescription reduction was similar, with an adjusted absolute difference of −45.0% (95% CI −56.3% to −33.6%) (Supplementary Table 4).

**Table 3:** Antibiotic prescription and clinical outcomes among sick children in the DYNAMIC trial

	ePOCT+, % (*n/N*)	Usual care, % (*n/N*)	Intracluster correlation coefficient (95% CI)	Crude difference (95% CI)	Adjusted difference (95% CI)	Crude relative risk (95% CI)	*P* value	Adjusted relative risk (95% CI)	*P* value
Primary outcome									
Antibiotic prescription at day 0	23.2% (3,806/16,381)	70.1% (12,058/17,205)	0.3 (0.2; 0.4)	−46.9% (−47.8%; −45.9%)	−46.4% (−57.6%; −35.2%)	0.33 (0.32; 0.34)	<0.001	0.35 (0.29; 0.43)	<0.001
Clinical failure by day 7	3.7% (532/14,396)	3.8% (543/14,363)	0.004 (0.001; 0.006)	−0.1% (−0.5%; 0.4%)	−0.1% (−0.6%; 0.3%)	0.98 (0.87; 1.10)	0.70	0.97 (0.85; 1.10)	0.59
Secondary and exploratory outcomes									
Death by day 7	0.1% (9/14,396)	0.1% (11/14,363)	<0.001	0.0% (−0.1%; 0.0%)	0.0% (−0.1%; 0.0%)	0.82 (0.34; 1.97)	0.65	0.66 (0.24, 1.84)	0.43
Subjectively worse at day 7^a^	0.3% (41/14,396)	0.3% (40/14,363)	0.002 (0.000; 0.004)	0.0% (−0.1%; 0.1%)	0.0% (−0.1%; 0.2%)	1.02 (0.66; 1.58)	0.92	1.11 (0.71; 1.73)	0.65
Nonreferred secondary hospitalizations by day 7	0.4% (57/14,396)	0.4% (50/14,363)	0.001 (0.000; 0.002)	0.0% (−0.1%; 0.2%)	0.0% (−0.0%; 0.2%)	1.14 (0.78; 1.66)	0.51	1.14 (0.77; 1.69)	0.52
Hospitalizations by day 7^a^	1.0% (145/14,396)	0.9% (130/14,363)	0.01 (0.01; 0.02)	0.1% (−0.1%; 0.3%)	0.3% (−0.0%; 0.7%)	1.11 (0.88; 1.41)	0.38	1.43 (1.00; 2.05)	0.05
Primary referrals at day 0	1.2% (194/16,381)	1.0% (170/17,205)	0.03 (0.01; 0.04)	0.1% (−0.2%; 0.3%)	0.8% (0.1%; 1.5%)	1.2 (0.98; 1.47)	0.08	2.08 (1.15; 3.74)	0.02
Referral resulting in hospitalization by day 7^b^	16.8% (25/149)	20.3% (29/143)	0.05 (0.00; 0.14)	−3.5% (−5.4%; 12.4%)	−2.8% (−11.8%; 6.2%)	0.83 (0.51; 1.34)	0.44	0.86 (0.53; 1.40)	0.55
Unplanned reattendance visits by day 7^c^	1.8% (256/14,603)	2.9% (425/14,723)	0.03 (0.01; 0.04)	−1.1% (−1.5%; −0.8%)	−1.0% (−2.8%; 0.9%)	0.61 (0.52; 0.71)	<0.001	0.67 (0.32; 1.44)	0.31
Additional medication taken after initial consultation up to day 7	7.1% (1,006/14,244)	7.2% (1,017/14,229)	0.006	−0.1% (−0.7%; 0.5%)	−0.9% (−2.1%; 0.4%)	0.99 (0.91; 1.07)	0.78	0.88 (0.74; 1.05)	0.17

All data shown for day 0 outcomes are per protocol, and all data for day 7 outcomes are per protocol and complete case (day 7 outcomes assessed). Clinical failure by day 7 defined as ‘not cured’ and ‘not improved’, or unscheduled hospitalization as reported by caregivers. Nonreferred secondary hospitalizations by day 7 are hospitalizations at least a day after the initial consultation that were not referred by a health-care provider. Unplanned reattendance visits by day 7 are return visits between day 1 and 7 that were not proposed by the initial health-care provider. Adjusted relative risks and differences were estimated using a random effects logistic regression model adjusting for clustering (health facility and patient), as well as individual (age, sex, complaints, availability of phone) and health facility (council of health facility, level of health facility, mean number of patients seen per month at the health facility) baseline characteristics. Formal adjustments were not performed for multiple testing.

^a^Post hoc exploratory outcome not prespecified.

^b^Denominator is based on consultations for which a primary referral was proposed and day 7 hospitalization data were ascertained, and as such may be less than the total number of primary referrals at day 0.

^c^Including unplanned outpatient and hospitalized reattendance visits.

**Fig. 3:**
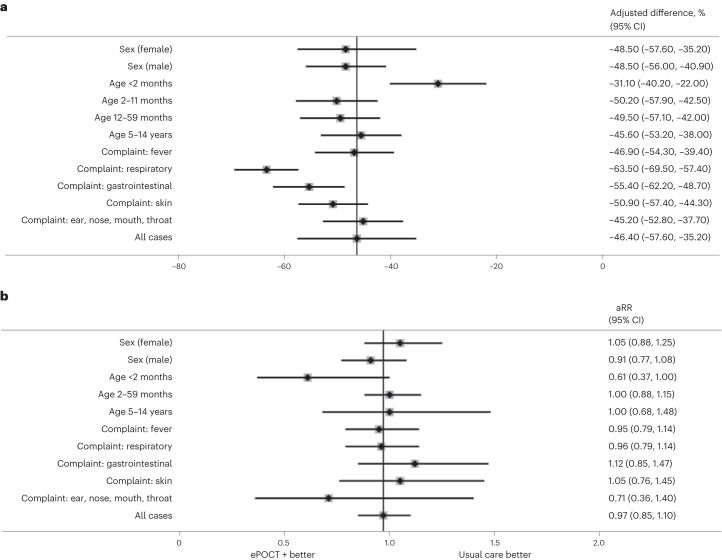
Antibiotic prescription and clinical failure by sex, age group and main complaints. **a**, Data are presented as adjusted differences with 95% CI of day 0 antibiotic prescription between ePOCT+ health facilities and usual care health facilities. All data are from the per protocol population in initial consultations. Sample sizes for each subgroup are found in Extended Data Table 2. **b**, Data are presented as adjusted relative risk with 95% CI of clinical failure in ePOCT+ compared to usual care health facilities. All data are from the per protocol and complete case population among initial consultations. Sample sizes for each subgroup are found in Extended Data Table 3.

The proportion of patients with clinical failure by day 7 was noninferior in ePOCT+ health facilities (3.7%, 532 of 14,396) compared to usual care health facilities (3.8%, 543 of 14,363), with an adjusted relative risk of 0.97 (95% CI 0.85 to 1.10) in the per protocol complete case population (Table 3 and Fig. 3). Clinical failure by day 7 was also noninferior in the intention-to-treat complete case population (Extended Data Table 1), when including reattendance cases (Supplementary Table 4) and using unadjusted analyses (Table 3 and Fig. 2).

### Secondary and exploratory clinical safety outcomes

There were no significant differences in the proportion of patients who died, were subjectively worse or, were hospitalized after the day of the initial consultation without a referral (nonreferred secondary hospitalization), all hospitalizations by day 7 or unplanned reattendance visits (Table 3). There was however a significant reduction in unplanned reattendance visits by day 7 in unadjusted analyses. The proportion of patients who died (0.1%) or were hospitalized (1.0%) was low in both study arms. Results in the intention-to-treat population and when including reattendance visits were similar (Extended Data Table 1 and Supplementary Table 4).

### Additional medications by day 7 and antibiotic prescription over time

At day 7 (range 6–14), additional medicines were taken after the initial consultation in a similar proportion of patients in both study arms (7.1% versus 7.2% in intervention versus control health facilities, Table 3). When evaluating evolution of mean antibiotic prescription rates over time, it appears to decrease over time in ePOCT+ health facilities, whereas no change was found in usual care facilities (Supplementary Fig. 1).

### Referral and hospitalizations

Health-care providers identified 3.6% (582 of 15,799) of cases as having a severe diagnosis in ePOCT+ facilities compared to 2.6% (453 of 17,205) in usual care facilities (per protocol in initial cases). The proportion of cases referred for hospitalization was higher in ePOCT+ facilities (1.2%) than in usual care facilities (1.0%) (aRR 2.08, 95% CI 1.15 to 3.74) (Table 3). The proportion of children referred that resulted in hospitalization was low and similar in both study arms (Table 3). The proportion of cases referred to specialized outpatient clinics (malnutrition clinic, tuberculosis investigation, HIV clinic) was low and similar between ePOCT+ and usual care health facilities (Supplementary Table 5).

### Subgroup analyses: sex, age, complaints

The effect of the intervention on antibiotic prescription at day 0 was more pronounced in children presenting with respiratory complaints (absolute difference −62.1%, 95% CI −63.3% to −60.9%) and the 2–59-month age group (absolute difference −48.9%, 95% CI −49.9% to −47.9%) (Fig. 3 and Extended Data Table 2). Antibiotic prescription was reduced by at least 25 percentage points in all prespecified subgroups, with the smallest reduction found in infants under 2 months old (absolute difference −25.5%, 95% CI −30.3% to −20.6%). Among post hoc subgroup analyses, patients with a positive malaria test had a lower reduction in antibiotic prescription between ePOCT+ and usual care (adjusted absolute difference −18.8%, 95% CI −25.1% to −12.6%) (Extended Data Table 2). Young infants less than 2 months old had the largest reduction in day 7 clinical failure (aRR 0.61, 95% CI 0.37 to 1.00; *P* = 0.05) (Fig. 3 and Extended Data Table 3).

## Discussion

In this cluster randomized controlled trial involving 44,306 sick children under 15 years of age in Tanzania, the use of the ePOCT+ digital clinical decision support algorithm (CDSA) package resulted in a close-to three-fold reduction in the likelihood of a sick child receiving an antibiotic prescription compared to children in usual care facilities. Despite substantially fewer antibiotic prescriptions, clinical failure did not increase in intervention facilities. Such findings align with Tanzania’s National Action Plan to reduce antibiotic use^17^ and are in line with the Tanzania digital health strategy to improve quality of care^32^.

The reduction of antibiotic prescription associated with the ePOCT+ intervention in our study is consistent with our previous research with CDSAs in Tanzania in more controlled research settings^20^^,21,33^. However, the results differ from other studies evaluating CDSAs implemented in routine health programs in Nigeria, Afghanistan, Burkina Faso and South-Africa, and in a controlled study setting in Uganda, which found smaller and even no reduction in antibiotic prescription^22–24^^,34^^,35^. There are a number of differences that may explain the divergent results. First and foremost, the clinical algorithm of ePOCT+ differs from other CDSAs. It notably has a wider scope including additional conditions and point-of-care tests such as C-reactive protein (CRP), not included in the Integrated Management of Childhood Illness (IMCI)^28^. A randomized controlled trial comparing two different CDSAs found differences in the impact of antibiotic stewardship due to the addition of CRP and other algorithm modifications, demonstrating that not all CDSAs are equal^20^. Other differences that may explain the divergent results include (1) differences in the supportive training and mentorship provided, (2) disease epidemiology (notably malaria prevalence) and (3) health-care provider skills and adherence. The extent of the impact on antibiotic stewardship in our study is also greater than that observed in other antibiotic stewardship studies that included one single intervention rather than an intervention package^36,37^. ePOCT+ integrates multiple proven antibiotic stewardship interventions together, including clinical decision support^20^^,21,33^, the use of point-of-care CRP tests^38^, pulse oximeter^39^ and continuous quality improvement mentorship support with data feedback to health-care providers utilizing benchmarking of health facilities^40,41^.

Clinical failure was not higher in patients managed in ePOCT+ health facilities, despite a significant reduction in antibiotic prescription in line with other antibiotic stewardship studies^38^. Similarly the proportions of children who died, were hospitalized without referral or had unplanned reattendance visits were not higher. Whereas previous CDSA studies were able to demonstrate significant reductions in clinical failure, the current trial was not powered to do so^20,21,23^. Nonetheless, the greatest benefit on clinical cure compared to usual care was observed in the subgroup of infants aged under 2 months, important results given that this population represents more than 50% of mortality in children under 5 years old^42^.

Although the present findings are encouraging, it is important to note that nearly 25% of patients were not managed using ePOCT+ in the intervention arm. Lower uptake of the tool could reduce the positive impact of antibiotic stewardship as seen in the lower reduction in antibiotic prescription in the intention-to-treat (ITT) population. It is reasonable to assume that not all health providers use the digital tool to manage all patients, just as health providers do not consult the IMCI paper chartbook every time they see a patient. Indeed CDSAs have been found to improve adherence to IMCI guidelines^23,24,43,44^, nonetheless many challenges in adherence to paper guidelines remain for digital tools, notably low motivation, lack of on-site mentoring and cognitive overload^45^^,46^. The use of electronic medical record (EMR) systems in some health facilities may also explain poor uptake, as some providers were expected to input clinical data in ePOCT+, the EMR and a paper log, prolonging the consultation time. Integration of clinical decision support within the EMR system instead of separate standalone systems could help and is currently being explored. In addition to harmonization of digital health tools, numerous other factors must be considered and are currently being evaluated in order for ePOCT+ and similar tools to be adequately scaled up in Tanzania and other countries. They include a better understanding of why health providers did not use ePOCT+ and how the clinical algorithms of ePOCT+ can be further improved, how health providers can be better supported to use the digital tools, the impact of benchmarking and mentoring dashboards, cost and greenhouse gas emission analyses, and acceptance by patients and community members.

Our study possesses several strengths that contribute to its robustness. First, we employed a cluster randomized controlled study design, which was adequately powered to assess noninferiority of clinical failure. Second, the implementation of our intervention encompassed a wide range of epidemiological settings, including both rural and urban areas, with varying levels of malaria transmission and facilities such as dispensaries and health centers. Moreover, our study employed comprehensive patient inclusion criteria that were designed to be inclusive. By incorporating these inclusive criteria, randomly sampling health facilities for inclusion, and observing consistent effects across subgroups at both the health facility and individual levels, our findings can be generalized to a broader population.

There are several limitations to our study. First, antibiotic prescription data relied on documentation by the health-care provider, an approach often used in pragmatic trials^47,48^. When using a conservative imputation analysis approach in the ITT population considering that all patients for which treatment was not documented were considered to have been prescribed an antibiotic, ePOCT+ still reduced antibiotic prescription considerably (Extended Data Table 1). Second, despite multiple phone calls and home visits, 15% of cases were lost to follow-up, consistent with data from similar studies (13–25%)^49–51^. To account for potential biases in loss to follow-up, we adjusted the final model for baseline variables associated with missing outcome data, analogous to performing multiple imputation in the case of a single endpoint. Third, the fact that a child has not improved after day 7 sometimes reflects the natural course of the disease, rather than the poor quality of care at the initial consultation, and may not therefore be expected to be influenced by the intervention for all clinical situations. To show an effect on more severe outcomes such as secondary hospitalization, death or even clinical failure at day 14 or 28 would require a very large sample size owing to the rarity of the event at the primary care level. Further complicating assessment of these severe outcomes are the challenges linked to referral and quality of care at admitting hospitals.

In conclusion, the ePOCT+ electronic clinical decision support algorithm (CDSA) in association with point-of-care tests (CRP, hemoglobin, pulse oximeter) and mentorship support informed by clinical practice data, safely and substantially reduced antibiotic prescription in sick children less than 15 years of age presenting to primary care facilities in Tanzania. Widespread implementation of ePOCT+ could help address the urgent problem of antimicrobial resistance by reducing excessive antibiotic prescription in sick children while maintaining clinical safety.

## Methods

### Study design and setting

The DYNAMIC Tanzania study was a pragmatic, open-label, parallel-group, cluster randomized trial conducted in 40 primary health facilities in Tanzania. The health facility was the unit of randomization, since the intervention was targeted at the health facility level.

Study sites were purposefully chosen to represent a variety of health-care and epidemiological settings within five councils in the Mbeya and Morogoro region, with a total population in those councils of 1,701,717 (ref. ^52^). Two councils were semiurban (Mbeya city and Ifakara Town councils), whereas the three others were rural (Mbeya, Ulanga and Mlimba district councils). Overall 42.8% of the Tanzanian population is less than 15 years old^53^. The prevalence of malaria in febrile children aged 6–59 months is 5.8% in the Morogoro region and 3.4% in the Mbeya region^54^. In accordance with the Tanzanian national clinical guidelines, all febrile patients should be tested for malaria using a rapid diagnostic test at the health facility of contact^55^. HIV prevalence among children less than 15 years old is 0.5% in both regions^56^. Health care for acute illnesses at government or government-designated primary health facilities is free of charge for children under 5 years, including the cost of medications such as antibiotics. For patients older than 5 years, health-care expenses are charged to the patient, unless they have a health insurance plan (around 10% of Tanzanians)^57^.

First-level health facilities included in the DYNAMIC Tanzania study include dispensaries and health centers with the latter distinguished by several characteristics. Health centers are characterized by multiple outpatient consultation rooms, potential presence of medical doctors, occasional small inpatient wards and a broader array of diagnostic and therapeutic capabilities compared to dispensaries.

### Participants

Primary care health facilities (dispensaries or health centers) were eligible for inclusion if they performed on average 20 or more consultations per week with children aged from 2 months to 5 years, were government or government-designated health facilities, and were located less than 150 km from the research institutions. Acute outpatient care is routinely provided by nurses and clinical officers in primary health facilities, whereas medical doctors provide care occasionally at health centers. Clinical officers, the principal health providers at primary health facilities, are non-physician health professionals with 2–3 years of clinical training following secondary school^58^.

Infants and children between 1 day old and 15 years old seeking care for an acute medical or surgical condition at participating health facilities were eligible. Children presenting solely for scheduled consultations for a chronic disease (for example HIV, tuberculosis, malnutrition) or for routine preventive care (for example growth monitoring, vaccination) were not eligible. Written informed consent was obtained from all parents or guardians of participants when attending the participating health facility during the enrollment period.

### Sampling, randomization and masking

The 40 health facilities were randomly selected from all eligible health facilities in the participating councils following a 3:2 ratio between health facilities from the Morogoro and Mbeya region (to include more health facilities in the higher malaria transmission area). In addition, to include a representative sample of health centers compared to dispensaries, four health centers per region were included.

The sampled health facilities were then randomized (1:1) to ePOCT+ (intervention) or usual care (control). Randomization was stratified by region, council, level of health facility (health center versus dispensary) and attendance rate. An independent statistician in Switzerland was provided with the list of all eligible health facilities and performed computer-generated sampling and randomization. Intervention allocation by the study team was only shared with study investigators in Tanzania once all council leaders had confirmed the participation of their selected health facilities. The nature of the intervention did not allow for masking of the intervention to health-care providers, patients or study implementers.

### Intervention

**Extended Data Fig. 1:**
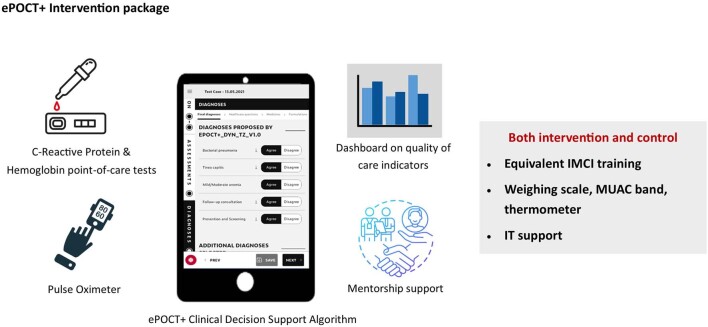
Description of ePOCT+ and supportive mentorship intervention. The intervention included the provision of the ePOCT+ Clinical Decision Support Algorithm (CDSA), C-Reactive Protein & Hemoglobin point-of-care tests, and pulse oximeter. The use of these additional tests and tools are proposed within the ePOCT+ CDSA. The intervention also included the sharing of quality of care indicators within dashboards which allowed healthcare providers to see their performance compared to other health facilities. Finally mentorship support in the form of messages, phone calls, and visits were conducted to answer questions and support the use of ePOCT+. Both intervention and control health facilities received equivalent Integrated Management of Childhood Illness (IMCI) training, and Information Technology (IT) support. If required the health facilities also received a weighing scale, mid-upperarm circumference (MUAC) band, and thermometer.

The intervention consisted of providing ePOCT+ with the supporting IT infrastructure, C-reactive protein (CRP) semiquantitative lateral flow test, hemoglobin point-of-care tests (and hemoglobinometer if not already available), pulse oximeter, training and supportive mentorship (Extended Data Fig. 1). If unavailable in health facilities, materials to perform laboratory tests such as prickers, cotton swabs, gloves and alcohol were provided. The decision to perform tests (malaria, CRP, hemoglobin, pulse oximeter), like all clinical symptoms and signs, is determined by the clinical algorithm behind ePOCT+, and prompted to the health-care provider when required. The health-care provider can decide not to follow the recommendations of ePOCT+ as they see fit. CRP point-of-care rapid tests and hemoglobin point-of-care tests were integrated as per usual laboratory procedures (that is, in health facilities where point-of-care tests are usually performed and interpreted in the laboratory by a laboratory technician, tests were performed in the laboratory; in health facilities where tests are usually done in the consultation room, they were done by the health-care provider). The development process and details of the ePOCT+ CDSA and the medAL-reader Android-based application used to deploy ePOCT+ have been described in detail previously^28^. In summary the clinical algorithm of ePOCT+ is based on previous-generation CDSAs (ALMANACH and ePOCT)^20^^,29^, international and national clinical guidelines, and input from national and international expert panels, and was adapted based on piloting and health-care provider feedback^28^. Mentorship by the implementation team included visits to health facilities every 2 to 3 months and communication by phone call or group messages three to four times per month, to resolve issues and provide guidance and feedback on the use of the new tools. Results from quality-of-care dashboards were shared through group messages to give feedback on the use of ePOCT+, a strategy often described as ‘benchmarking’, allowing health-care providers to compare their antibiotic prescription, uptake and other quality-of-care indicators with other health facilities^59^. Control health facilities provided care as usual, with no access to clinical data dashboards.

All participating health facilities were provided with IT infrastructure to support the tablet-based ePOCT+ CDSA or in the case of control health facilities, to support the use of tablet-based eCRFs. The IT infrastructure included a tablet for each outpatient consultation room, router, local server (Rasberry Pi), internet and, if needed, back up power (battery) or solar power. In addition, weighing scales, mid-upper arm circumference bands and thermometers were provided to health facilities for both study arms if not already available. Health-care providers from both intervention and control health facilities received equivalent clinical refresher training based on the IMCI chartbook. In addition, specific training was provided on the use of the ePOCT+ CDSA in intervention facilities and the use of the eCRF in control facilities.

### Study procedures

Children seeking care at included health facilities were screened for eligibility by a research assistant between 08:00 and 16:00 on weekdays. If eligible, demographic information was collected and entered in the eCRF (ePOCT+ for intervention health facilities and eCRFs for usual care facilities within the data collection system medAL-reader). Health-care providers in the control health facilities managed the patients as usual, but documented the main complaints, anthropometrics and test results (if performed), diagnoses, treatments and referral decision in the eCRF. To harmonize data collection across the intervention and control facilities, the eCRF for the control facilities was also programmed into the medAL-reader platform, but no decision support was provided. Research questions were included in the eCRF to capture whether an oral or systemic antibiotic was prescribed, and whether the patient was referred for inpatient hospitalization or other outpatient investigations. In intervention health facilities, in addition to the same information collected in the eCRF, symptoms and signs of the patients were recorded in the ePOCT+ CDSA during the consultation with the patient. The symptoms and signs entered are used by the ePOCT+ CDSA to guide the clinical consultation. Health-care providers who documented the final treatment for a consultation in ePOCT+ or the eCRF were categorized as having been managed per protocol, as recording of the final treatment is required to complete the ePOCT+ CDSA.

All patients were called or visited at their home by research assistants to assess clinical outcomes and their care and treatment seeking behavior at day 7 (range 6–14 days). Research assistants performing the phone calls were blinded to the intervention status and were not part of the team enrolling patients at health facilities. Home visits rather than phone calls were conducted if the caregiver of patients did not have a phone number or did not know somebody with a phone near their home, or if research assistants were not able to reach the provided phone number after five attempts. The home visits were performed by the research assistants enrolling patients from the same health facility, and as such they were not blinded to intervention allocation. Patients who were still sick at follow-up were encouraged to return to a health facility for follow-up care. Day 7 data were recorded using REDCap web for phone calls and REDCap mobile application for home visits.

### Outcomes

The coprimary outcomes measured at the individual patient level included: (1) antibiotic prescription at the time of the initial consultation as documented by the health-care provider (superiority analysis); and (2) clinical failure at day 7 defined as ‘not cured’ and ‘not improved’, or unscheduled hospitalization as reported by caregivers (noninferiority analysis). Secondary outcomes include unscheduled reattendance visits at any health facility by day 7, nonreferred secondary hospitalization by day 7, death by day 7 and referral for inpatient hospitalization at initial consultation. Additional antibiotics prescribed on subsequent days following the initial consultation were not part of the coprimary outcome of antibiotic prescription; instead this is captured by phone call on day 7, where all patients are assessed for whether additional medication was taken after the initial consultation, and compared between study arms as an exploratory outcome. Given patients’ and caregivers’ difficulty in distinguishing antibiotics from other medications^60,61^, we could not reliably assess antibiotic intake based on the caregiver’s report; the outcome thus looked at all medications, rather than antibiotics specifically. The intervention was deemed a success if ePOCT+ was noninferior in terms of clinical failure and reduced antibiotic prescription by at least 25%. Prespecified additional outcomes are outlined in the statistical analysis plan.

### Sample size

The sample size was calculated for testing noninferiority of the clinical failure outcome given that it would require a higher sample size than for the antibiotic prescription coprimary outcome. We assumed a cluster size of 900 patients per health facility (mean of 150 patients per month per health facility multiplied by 6 months, the minimum duration of the study) based on routine data within the national health management information system, an intraclass correlation coefficient of 0.002 and a clinical failure rate of 3%. To have 80% power to detect an acceptable noninferiority margin of a relative risk of 1.3, corresponding to 3.9%, we required 19 clusters and 17,100 patients per arm (total patients *n* = 37,620 assuming 10% loss to follow-up). Given the uncertainty of some of the assumptions, the total number of health facilities was rounded up to 20 clusters per arm.

No interim analysis was planned; however, owing to lower enrollment than expected, after 8 months of recruitment, we planned an ad hoc sample size recalculation by an independent statistician to calculate the expected power of the study based on updated parameters (Supplementary Information Note 1). The study team prespecified the specifications and approach, documented in an update to the statistical analysis plan.

### Statistical analysis

All outcomes were evaluated using random effects logistic regression models using the cluster (health facility) and patient as random effects, with further adjustment using fixed effect terms for randomization stratification factors^62^, and baseline characteristics hypothesized to be associated with the outcome, imbalances between arms and imbalances between characteristics among patients for whom day 7 data were available and not available (lost to follow-up). These included the patient characteristics of age, sex, presenting complaints (fever, respiratory, gastrointestinal, skin) and phone availability, and the health facility characteristics of care provision level (dispensary versus health center), attendance rate per month and council. A partitioning method was used to separate within-cluster and between-cluster effects to account for confounding by cluster^63–65^. In the case of too few events, and small variance among health facilities, which did not allow the model to converge, the health facility was incorporated in the model as a fixed effect. Adjusted relative risk and absolute differences were estimated based on the computed marginal probabilities of the conditional probabilities^66^^,67^. Formal adjustments were not performed for multiple testing, as adjustments would likely be overly conservative given that the outcomes are not all independent^68^, and variable selection was not based on statistical tests of significance^69^. No adjustment for baseline characteristics or for within-health-care-facility correlations was used for the calculation of crude confidence intervals for relative risk and absolute differences.

Noninferiority was determined if the upper limit of the 95% CI of the aRR was below 1.3. All analyses based on outcomes from day 0 were performed in the per protocol population, and outcomes determined at day 7 were performed in the per protocol and complete case population (only in those for which day 7 outcomes were ascertained) and displayed accordingly unless stated otherwise. The primary analyses were performed on the first visit for an illness, with reattendance visits (a second visit to a health facility for the same illness) included in exploratory analyses. Prespecified analyses to assess the effect of the intervention in different population groups were performed by sex, age group and consultation complaint categories (respiratory symptoms, fever, gastrointestinal complaint, skin problem, ear, nose and throat problem). All analyses were performed using Stata v.16 and v.17 (ref. ^70^).

### Inclusion and ethics

Ethical approval was obtained in Tanzania from the Ifakara Health Institute (IHI/IRB/No: 11-2020), the Mbeya Medical Research Ethics Committee (SZEC-2439/R.A/V.1/65) and the National Institute for Medical Research Ethics Committee (NIMR/HQ/R.8a/Vol. IX/3486 and NIMR/HQ/R.8a/Vol. IX/3583), and in Switzerland from the cantonal ethics review board of Vaud (CER-VD 2020-02800). The study was registered on ClinicalTrials.gov number NCT05144763, where the trial protocol and statistical analysis plan can be found (statistical analysis plan also found in Supplementary Information Note 2). The study design and implementation was developed collaboratively between the Ifakara Health Institute, Mbeya Medical Research Centre, Swiss Tropical and Public Health Institute and the Centre for Primary Care and Public Health, University of Lausanne, based on feedback from stakeholders, patients and health-care providers involved in our similar trials in Tanzania^20^^,21,33^. In addition, previous work from Tanzania was used to guide the design of the study and to develop ePOCT+, and other work from Tanzania was taken into account in the citations for this manuscript^32^^,45^^,46^. ePOCT+ and the medAL-suite was developed collaboratively by an international group of digital and global health experts from Tanzania and other LMICs^28^. Specifically a Tanzanian clinical expert group including representatives designated by the Ministry of Health made the final decision on clinical content, and primary care level health providers gave important feedback to develop and improve ePOCT+, including a Delphi survey among 30 Tanzanian health providers^28^. Over 100 community engagement meetings with over 7,000 participants were conducted before and during the study, including numerous meetings with Community and Regional Health Management Teams in the Mbeya and Morogoro regions of Tanzania.

### Reporting summary

Further information on research design is available in the Nature Portfolio Reporting Summary linked to this article.

## Online content

Any methods, additional references, Nature Portfolio reporting summaries, source data, extended data, supplementary information, acknowledgements, peer review information; details of author contributions and competing interests; and statements of data and code availability are available at 10.1038/s41591-023-02633-9.

### Supplementary information

Supplementary Information Supplementary Tables 1–5, Fig. 1 and Notes 1 and 2.

Reporting Summary

## Figures and Tables

**Extended Data Table 1:**
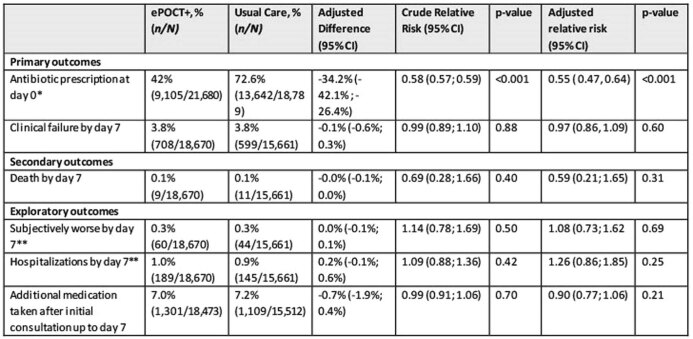
Primary, secondary and exploratory outcomes in initial cases in the intention-to treat population

Secondary and exploratory outcomes that rely on data collected on day 0 are not shown as this information is not available for all ITT patients (non-referred secondary hospitalizations by day 7, completed referral, primary referrals at day 0). All outcomes that rely on day 7 outcomes are complete case analyses. Adjusted relative risks and differences were estimated using a random effects logistic regression model adjusting for clustering (health facility, and patient), and individual and health facility baseline characteristics that were available for all ITT cases (age, sex, availability of phone, council of health facility, level of health facility, average number of patients seen per month at the health facility). Formal adjustments were not performed for multiple testing. *Considering that all cases that were not managed per protocol were prescribed an antibiotic; **Post-hoc exploratory outcomes

**Extended Data Table 2:**
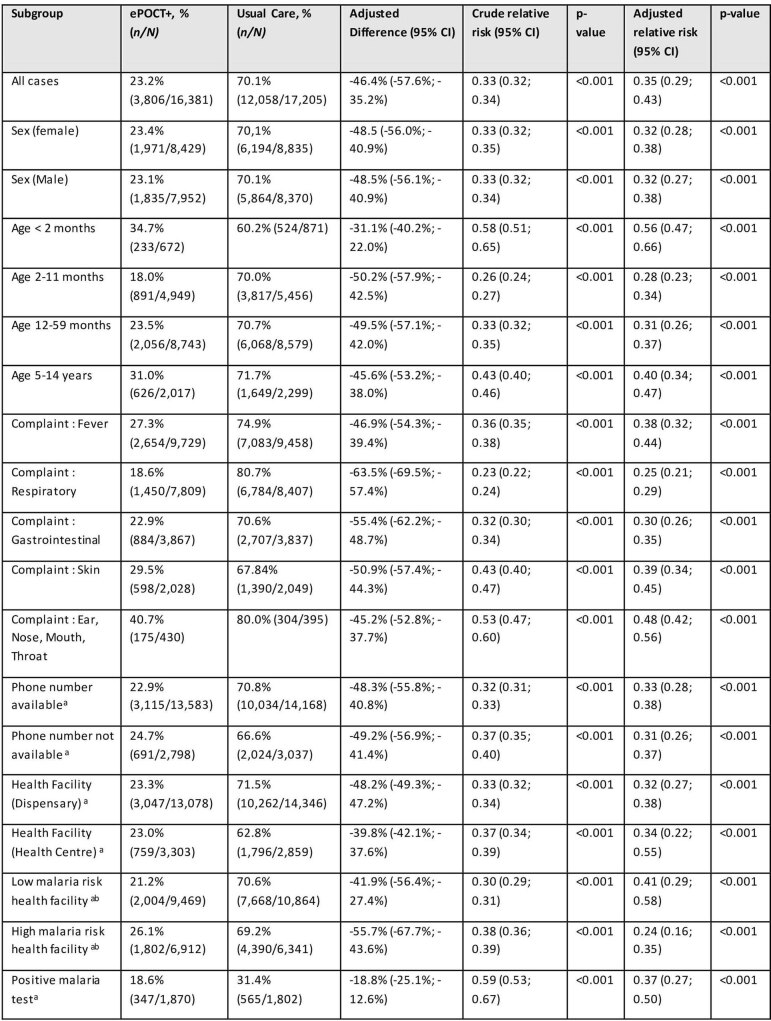
Stratified subgroup analysis for antibiotic prescription at D0 in initial consultations in the per protocol population

Adjusted relative risks and differences were estimated using a random effects logistic regression model adjusting for clustering (health facility and patient), and individual and health facility baseline characteristics (age, sex, complaints, phone availability, council of health facility, level of health facility, average number of patients seen per month at the health facility). Formal adjustments were not performed for multiple testing.

^a^Post-hoc exploratory subgroup analyses

^b^Malaria risk defined as per IMCI: Low risk = <5% malaria positivity rate in febrile children; High risk > = 5% malaria positivity rate in febrile children

**Extended Data Table 3:**
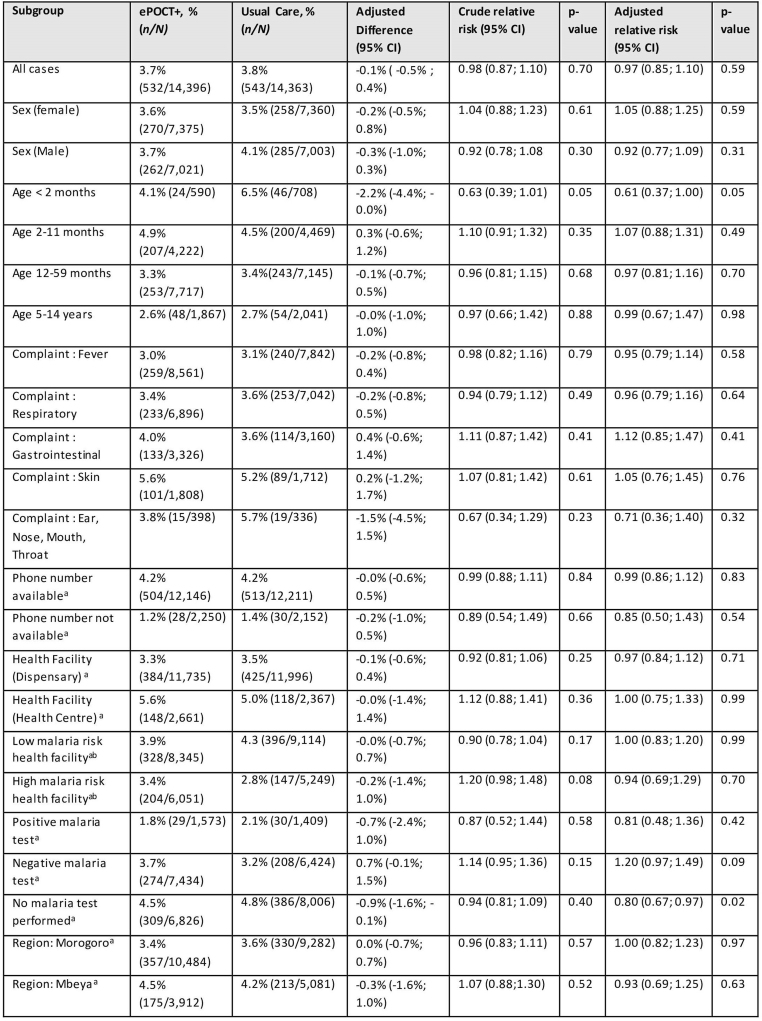
Subgroup analysis for clinical failure at day 7 in the per protocol, complete case population among initial consultations

Clinical failure by day 7 defined as ‘not cured’ and ‘not improved’, or unscheduled hospitalization as reported by caregivers. Adjusted relative risks were estimated using a random effects logistic regression model adjusting for clustering (health facility and patient), and individual and health facility baseline characteristics (age, sex, complaints, availability of phone, council of health facility, level of health facility, average number of patients seen per month at the health facility) but omitting the variable as an adjustment variable in the model for the subgroup analyzed. Formal adjustments were not performed for multiple testing.

^a^Post-hoc exploratory subgroup analyses

^b^Malaria risk defined as per IMCI: Low risk = <5% malaria positivity rate in febrile children; High risk > = 5% malaria positivity rate in febrile children

## Data Availability

De-identified data can be found at 10.5281/zenodo.8043523, including case, patient and health facility identification number, study arm allocation, baseline characteristics and all outcomes.

## Code availability

The code for the medAL-reader application used to collect data entered by health providers (including demographic, clinical, diagnosis, prescription and referral data of the consultations) can be found at https://github.com/Wavemind/liwi-medal-reader.
