# Supplementary material for: A digital health algorithm to guide antibiotic prescription in pediatric outpatient care: a cluster randomized controlled trial
Source: Nat Med. 2023 Dec 18;30(1):76–84. doi: 10.1038/s41591-023-02633-9 (PMC10803249; doi:10.1038/s41591-023-02633-9)
Supplement: Supplementary file 1 — Supplementary Tables 1–5, Fig. 1 and Notes 1 and 2. [file 41591_2023_2633_MOESM1_ESM.pdf]

nature medicine

Article https://doi.org/10.1038/s41591-023-02633-9

A digital health algorithm to guide antibiotic prescription in pediatric outpatient care: a cluster randomized controlled trial

In the format provided by the authors and unedited

Supplementary material

Table of Contents

Table S1: Baseline characteristics of enrolled participants lost to follow-up compared to not being lost to follow-up (complete case) in the per-protocol population ... 2

Table S2: Presenting complaints of enrolled participants lost to follow-up compared to not beinglost to follow-up (complete case) ... 3

Table S3: Baseline characteristics of all enrolled participants managed per protocol compared to those not managed per protocol ... 4

Table S4: Primary and secondary outcomes for all consultations (initial and re-attendance visits) in the per-protocol and complete case population ... 5

Table S5: Referral to specialized outpatient clinics in initial cases of the per protocol population ... 6

Figure S1: Mean antibiotic prescription over time of enrollment ... 7

Note S1: Sample size recalculation ... 8

Note S2: Statistical Analysis Plan ... 9

Table S1: Baseline characteristics of enrolled participants lost to follow-up compared to not being lost to follow-up (complete case) in the per-protocol population

	**Complete case %**	**Lost to follow-up %**
Number of cases, % (*n)*	85.8% (31,668)	14.2% (5,254)
Sex (female), % (*n)*	51.2% (16,218)	52.2% (2,742)
Age group, % (*n)*		
0 to <2 months	4.3% (1,360)	5.0% (265)
2 to <12 months	30.3% (9,606)	35.7% (1,875)
12 to <60 months	52.1% (16,485)	50.8% (2,670)
5 to <15 years	13.3% (4,217)	8.5% (444)
Type of consultation, % (*n)*		
New consultation	90.8% (28,759)	91.9% (4,827)
Follow-up	8.9% (2830)	8.0% (419)
Referral from another health facility	0.3% (79)	0.2% (8)
Hospitalized in the last 14 days, % (*n)*	0.3% (101)	0.4% (23)
Availability of phone, % (*n)*	85.0% (26,909)	70.9% (3,725)
Level of health facility, % (*n)*		
Health centre	18.9% (6,108)	23.7% (1,097)
Dispensary	81.1% (26,185)	76.3% (3,532)

Table S2: Presenting complaints of enrolled participants lost to follow-up compared to not beinglost to follow-up (complete case)

**Presenting complaints in <2 months**	**Complete case, % (*N*=1,360)**	**Lost to follow-up, % (*N*=265)**
Fever, convulsions, lethargy	18.2%	20.0%
Respiratory	46.0%	44.2%
Gastrointestinal	21.1%	20.7%
Skin	14.3%	12.8%
Ear/mouth	1.7%	0.8%
Eye	6.5%	3.8%
Feeding / weight	0.6%	0.8%
Malformation	0.3%	1.1%
Injuries	0.3%	0%
Other	6.8%	9.4%
**Presenting complaints in >=2 months to <15 years**	**Complete case, % (*N*=31,668)**	**Lost to follow-up, % (*N*=5,254)**
Fever	56.5%	56.9%
Respiratory (cough / difficulty breathing	46.6%	45.1%
Gastrointestinal (diarrhea/vomiting)	21.5%	23.9%
Skin	11.8%	10.9%
Ear/throat/mouth	2.4%	1.8%
Eye	2%	1.9%
Genitourinary (UTI, STI)	2.2%	1.7%
Neurological (Headache, stiff neck)	2.1%	1.7%
Accident / muskuloskeletal (incl. burns, wounds, poison)	1.7%	1.6%
Other	3.1%	2.8%

Table S3: Baseline characteristics of all enrolled participants managed per protocol compared to those not managed per protocol

	Per protocol	Not per-protocol
Number, % (*n*)	83.3% (36,922)	16.7% (7,384)
Sex (female), % (*n*)	51.4% (18,960)	50.8% (3,748)
Age group, % (*n*)		
0 to <2 months	4.4% (1,625)	4.7% (343)
2 to <12 months	31.1% (11,481)	33.1% (2,444)
12 to <60 months	51.9% (19,155)	51.1% (3,775)
5 to <15 years	12.6% (4,661)	11.1% (822)
Type of consultation, % (*n*)		
New consultation	91% (33,586)	93,2% (6,883)
Follow-up	8.8% (3249)	6.7% (491)
Referral from another health facility	0.2% (87)	0.1% (10)
Hospitalized in the last 14 days, % (*n*)	0.3% (124)	0.2% (14)
Availability of phone, % (*n*)	83.0% (30,634)	86.1% (6,360)
Level of health facility, % (*n*)		
Health centre	19.5% (7,205)	31.5% (2,328)
Dispensary	80.5% (29,717)	68.5% (5,056)

Table S4: Primary and secondary outcomes for all consultations (initial and re-attendance visits) in the per-protocol and complete case population

	ePOCT+, % (*n/N*)	Usual Care, % (*n/N*)	Adjusted difference (95% CI)	Crude relative risk (95% CI)	p-value	Adjusted relative risk (95% CI)	p-value
**Primary outcomes**							
Antibiotic prescription at day 0	23.4% (4,215/17,985)	69.7% (13,197/18,937)	-45.0% (-56.3%; -33.6%)	0.34 (0.33; 0.35)	<0.001	0.36 (0.31; 0.44)	<0.001
Clinical failure at day 7	3.9% (612/15,832)	3.9% (624/15,836)	-0.2% (-0.7%; 0.3%)	0.98 (0.88; 1.09)	0.73	0.95 (0.84; 1.07)	0.41
**Secondary and exploratory outcomes**							
Death by day 7	0.1% (11/15,832)	0.1% (12/15,836)	-0.0% (-0.1%; 0.1%)	0.92 (0.40; 2.08)	0.84	0.73 (0.29; 1.83)	0.51
Subjectively worse at day 7*	0.3% (47/15,832)	0.3% (45/15,836)	0.0% (-0.1%; 0.2%)	1.04 (0.69;1.57)	0.83	1.12 (0.74; 1.71)	0.59
Non-referred secondary hospitalizations by day 7	0.5% (76/15,832)	0.4% (59/15,836)	0.1% (-0.0%; 0.2%)	1.29 (0.92; 1.81)	0.14	1.26 (0.89;1.80)	0.19
Hospitalizations by day 7*	1.2% (189/15,832)	1.0% (159/15,836)	0.4% (-0.0%; 0.8%)	1.19 (0.96; 1.47)	0.11	1.39 (0.97; 2.0)	0.08
Completed referral (Hospitalization by day 7 if referred)	23.4% (43/184)	20.8% (33/159)	4.3% (-4.3%; 12.8%)	1.13 (0.75; 1.68)	0.56	1.21 (0.82; 1.80)	0.33
Primary referrals at day 0	1.3% (233/17,985)	1.0% (187/18,937)	0.2% (-0.1; 0.5%)	1.16 (0.94; 1.43)	0.17	1.19 (0.93; 1.53)	0.17
Uplanned re-attendance visits by day 7	1.8% (288/15,832)	3.1% (495/15,836)	-0.9% (-3.1%; 0.1%)	0.58 (0.50; 0.67)	<0.001	0.71 (0.32; 1.56)	0.39
Additional medication taken after initial consultation up to day 7	7.4% (1,153/15,634)	7.5% (1,169/15,673)	-0.8% (-2.2%; 0.5%)	0.99 (0.91; 1.07)	0.78	0.89 (0.74; 1.06)	0.20

Table legend: All outcomes that rely on day 7 outcomes are complete case analyses. Adjusted relative risks and differences were estimated using a random-effects logistic regression model adjusting for clustering (health facility, and patient), and individual and health facility baseline characteristics (age, sex, complaints, availability of phone, council of health facility, level of health facility, average number of patients seen per month at the health facility). Formal adjustments were not performed for multiple testing. *Post-hoc exploratory outcomes

Table S5: Referral to specialized outpatient clinics in initial cases of the per protocol population

	**ePOCT+, % (n)**<br>***N*=14,602**	**Usual Care, % (n)**<br>***N*=14,723**
Malnutrition clinic	0.1% (10)	0.0% (4)
Tuberculosis investigations	0.0% (5)	0.0% (0)
HIV clinic	0.1% (16)	0.1% (9)
Any referral to specialized outpatient clinics	0.2% (31)	0.1% (13)

Figure S1: Mean antibiotic prescription over time of enrollment

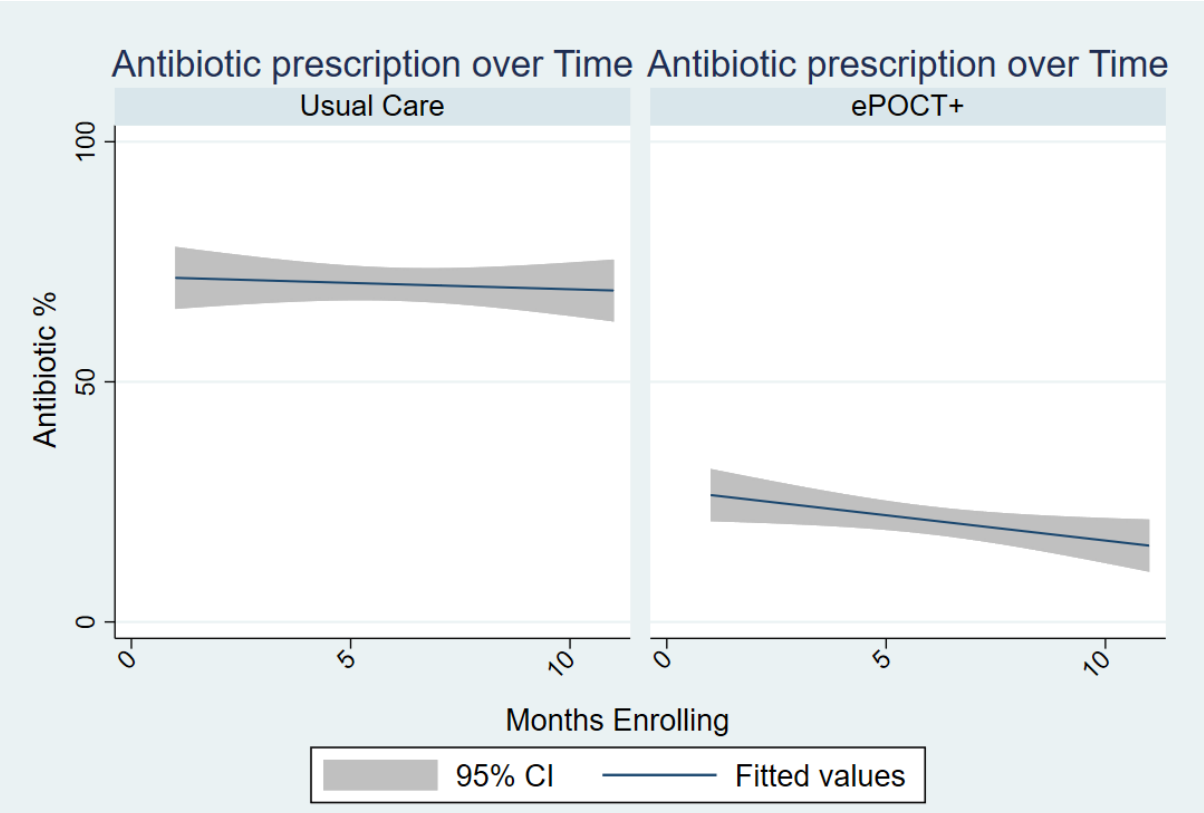

Figure legend S1: Center values are defined as the mean antibiotic prescription with 95% CI shown in the grey area. All data presented from the per protocol and initial consultation population.

Note S1: Sample size recalculation

At the time of sample size recalculation, 11 September 2022, using enrollment data up to 31 August 2022, the estimated average number of patients seen per health facility by mid-October 2022 (corresponding to the end of the minimum 6 months of enrollment in all health facilities) was calculated to be 741 patients. The independent statistician found the actual proportion of clinical failure to be 3.5% in the control arm. Using the actual proportion of clinical failure, the estimated number of patients up to mid-October 2022, and maintaining the other sample size parameters, it was found that we would have 100% power if the study were to end mid-October 2022. The study team was informed that it would have more than 80% power, and thus decided to plan the end of the study on 31 October 2022, leaving a small margin of error. Of note, the proportion of clinical failure in the control arm or the exact power of the sample size recalculation was not shared with the study team at the time of the sample size recalculation.

Note S2: Statistical Analysis Plan

DYNAMIC Tanzania Phase I Statistical Analysis Plan

**Trial registration number**	NCT05144763		
**SAP version number**	Version 1.2	**Date**	7 September 2022
**Protocol version**	This document has been written based on information contained in the study protocol version 5.0, dated 7 September 2022		

Authors	Position	Signature	Date
Rainer Tan	Research physician / clinical coordinator		7 September 2022
Alexandra Kulinkina	Project Coordinator		7 September 2022
Valérie D'Acremont	Principal Investigator		7 September 2022
**Approved by**			
Nyanda N. Elias	National Co-principal Investigator	DocuSigned by: 6F908436CC73443...	12-Sep-2022 | 02:46:36 PDT
Honorati Masanja	National Principal Investigator	DocuSigned by: 728BE860CB8A417...	09-Sep-2022 | 10:41:39 PDT

Contributing collaborators/authors

Name	Position
Irene Masanja	Scientific project leader
Chacha Mangu	Mbeya site investigator
Lameck Luwanda	Morogoro site investigator
Kristina Keitel	Scientific project leader
Gillian Levine	Neonatal epidemiologist
Mary-Anne Hartley	Scientific project leader
Sabine Renggli	Senior Research Scientist

Revision history

Protocol version	SAP version no.	Section number changed	Description of and reason for change	Date changed
v5.0	v1.2	3.4	Re-calculation of sample size due to lower than expected recruitment	7 September 2022

Contents

1. Abbreviations and glossary of terms 3
2. Introduction 4
   2.1 Hypothesis 4
   2.2 Objective 4
3. Methods 4
   3.1 Study design 4
   3.2 Randomization 6
   3.3 Sample size 6
   3.4 Statistical Interim analyses 6
   3.5 Timing of final analysis 7
4. Statistical principles 7
   4.1 Adherence and protocol deviations 7
   4.2 Analysis population definitions 7
5. Study population 7
   5.1 Screening data 7
   5.2 Eligibility 7
      5.2.1 Health facility (cluster) eligibility 7
      5.2.2 Study population eligibility 8
   5.3 Recruitment 8
   5.4 Baseline characteristics 9
6. Main analyses 10
   6.1 Outcome definitions 10
      6.1.1 Co-primary outcomes 10
      6.1.2 Secondary outcomes 10
   6.2 Analysis methods 11
   6.3 Exploratory analyses 12

References 15

1. Abbreviations and glossary of terms

CDSA	Clinical Decision Support Algorithm
CI	Confidence interval
CRP	C-reactive protein
ePOCT+	Name of CDSA (not an abbreviation)
HIV	Human Immunodeficiency Virus
HF	Health facility
HCW	Health care worker
IHI	Ifakara Health Institute
IQR	Interquartile range
IMCI	Integrated management of childhood illness
NCD	Non-communicable disease
NIMR	National Institute of Medical Research
PCs	Personal Computers
PHC	Primary health care
POC	Point-of-care
PRECIS-2	Pragmatic Explanatory Continuum Indicator Summary 2
RDT	Rapid Diagnostic Test
Swiss TPH	Swiss Tropical and Public Health Institute
TZ	Tanzania
TB	Tuberculosis
Unisanté	Centre for Primary Care and Public Health (University of Lausanne)
WHO	World Health Organization

2. Introduction

2.1 Hypothesis

Hypothesis: In routine conditions, Health Care Workers (HCWs) provided with an electronic clinical decision support algorithm (CDSA) on tablets or PCs can treat sick children attending primary health care facilities in Tanzania with appropriate antimicrobial prescriptions while maintaining a good clinical outcome.

Research hypothesis: The use of ePOCT+ for case management of sick children in primary care health facilities, compared to routine care, are prescribed less antibiotics at the initial consultation, and have the same proportion of cure rates at day 7 after their initial consultation.

2.2 Objective

Overall project objective: Improve the **integrated management of children with an acute illness** through the provision of an electronic CDSA (ePOCT+) to clinicians working at primary care level.

Objective of Phase 1 study:
The primary objective of this trial is to determine if the use of ePOCT+ for case management of sick children in primary care, compared to routine care:

(i) results in less antibiotic prescriptions at initial consultation;
(ii) is non-inferior in clinical outcomes at day 7.

3. Methods

3.1 Study design

The DYNAMIC TZ Phase 1 study is a pragmatic, open-label cluster randomized controlled study. The intervention evaluated consists of the provision to health workers the ePOCT+ clinical decision support algorithm (CDSA) displayed on tablets (medAL-reader), point-of-care tests proposed by ePOCT+ that are not part of routine care (pulse oximeter, CRP rapid test, additional hemoglobin cuvettes), complementary training on the tool, regular monitoring and mentorship/supervision visits by the study team and/or the Council Health Management Team (CHMT). Mentorship and supervision will be enabled by a complementary dashboard (medAL-monitor), used to visualize and monitor study-related indicators. Due to the pragmatic nature of the study, the design is adaptive, in that changes in the implementation throughout Phase 1 may be made based on monitoring data and feedback from the health facilities. These implementation changes (excluding significant adaptations to algorithm content) will be thoroughly documented and accounted for in longitudinal analyses.

Since the intervention takes place at the health provider level and their practices are influenced by the context of the health facility they are working in, randomization at health facility level rather than at HCW level or at individual patient level was chosen. The randomization of clusters (i.e. health facilities) will be performed on a 1:1 ratio. Thus, ePOCT+ will be provided to health care workers of 20 of the 40 selected primary health care facilities (intervention arm) in the Morogoro and Mbeya regions, while the other 20 health facilities (HFs) will serve as controls (control arm). Sick children aged 1 day to 14 years (inclusive) will be managed in accordance with the study arm to which the HF they attend has been assigned. The comparator for Phase 1 will be routine care as currently implemented in Tanzania supplemented with equivalent clinical training given to the intervention arm. This includes routine clinical supervision visits performed by CHMT members. To assess the primary and secondary outcomes, final decision on referral, laboratory test results, diagnoses and treatments at initial consultation will be recorded by HCWs electronically in both arms (in the intervention arm integrated into the CDSA; in the control arm via a simple eCRF without any decision support); children will also be followed-up by a phone call or home visit to their caregiver at day 7 (range 6-14) to determine their clinical status. The study is considered as pragmatic since there are no major exclusion criteria to the population of interest, and the setting and organization of the health facilities remains mostly the same (see PRECIS-2 wheel below).

The trial design is summarized below:

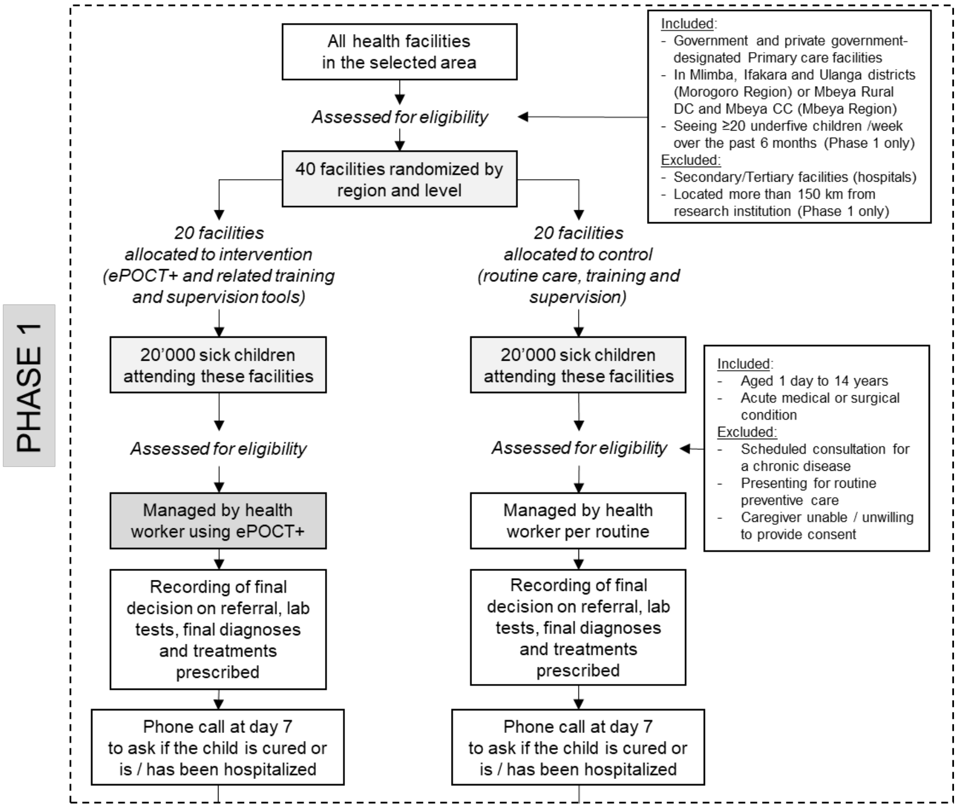

PRECIS-2 (Pragmatic-Explanatory Continuum Indicator Summary 2) wheel summarizing the extent of pragmatism of the study:

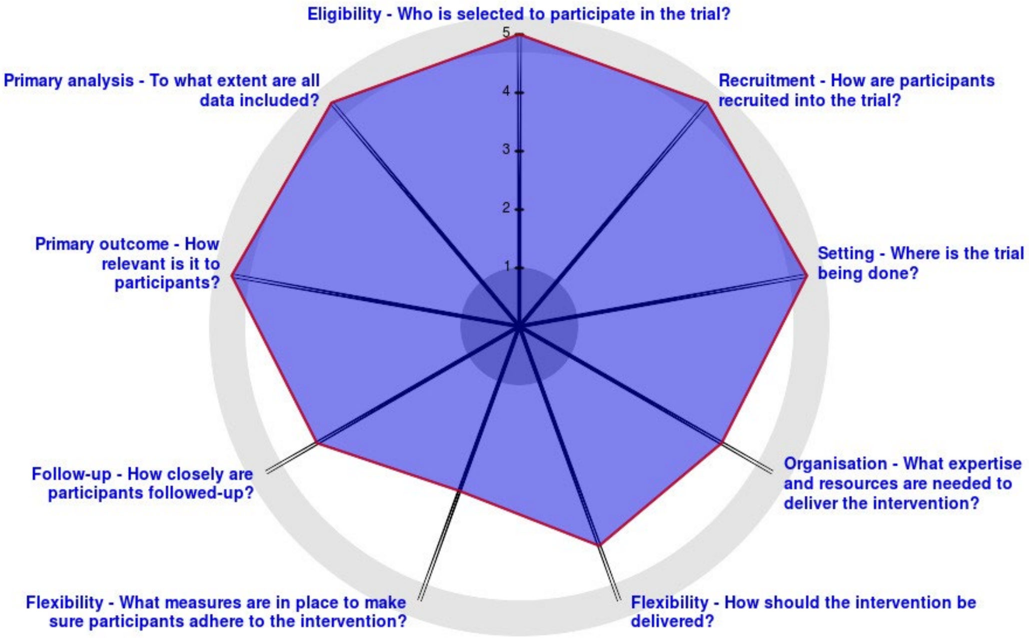

3.2 Randomization

The 40 health facilities to be included in the study will be randomly selected from all eligible health facilities in the participating councils (Mlimba district council, Ulanga district council, Ifakara town council, Mbeya district council, and Mbeya city council). Eligible health facilities include public health centers and dispensaries with at least 20 consultations with children between 2 months and 5 years of age per week. Cluster inclusion and exclusion criteria will first be applied to the full list of health facilities in the participating councils prior to selection and randomization. The random selection will follow a 3:2 ratio of health facilities between Morogoro and Mbeya regions, respectively, and include exactly 4 health centers per region.

Health facilities (or clusters) will be randomized (1:1), to using ePOCT+ (intervention) or routine care (control group). Randomization will be stratified by council, type of health facility (health center versus dispensary), and attendance rate. An independent statistician will perform both the sampling and randomization.

3.3 Sample size

The primary analysis will evaluate whether the use of ePOCT+ for case management of sick children in PHC facilities 1) results in similar clinical cure at day 7 (non-inferiority analysis), as compared to routine care and 2) decreased antibiotic prescriptions (superiority analysis) at the initial consultation. The sample size was calculated for testing the non-inferiority of the clinical cure outcome, which will require a much larger sample size due to the rareness of clinical failure. In addition, the testing of differences in antibiotic prescription is conditional on satisfying non-inferiority in the clinical outcome. Assuming a failure rate (not being cured at day 7) of 3% in the control arm (corresponding to a 97% clinical cure rate), based on previous studies, the acceptable non-inferiority margin will be a risk ratio of 1.3. This corresponds to the upper bound of the difference in failure rate not exceeding 0.9% if the control group failure rate is 3%. We utilized a fixed risk ratio rather than an absolute risk difference to protect against potential unexpected event rates (Head et al. 2012).

We assumed a cluster size of 900 patients (average of 150 patients per month x 6 months) and an intraclass correlation coefficient of 0.002 in the clinical outcome based on similar studies. To have 80% power to detect a risk ratio 1.3, for a one-sided hypothesis test at alpha of 0.025, we would require 19 clusters and 17,100 patients per arm (total patients n=37,620 assuming 10% loss to follow-up), rounded to a total of 40 HFs and 40’000 patients. However, due to uncertainty regarding the assumptions of the sample size calculation and to capture a wider more representative range of conditions, we will continue recruitment for Phase 1 for a minimum of 6 months.

3.4 Statistical Interim analyses

No formal interim analysis was planned for the primary endpoints. However due to lower enrollment than expected after 8 months of recruitment, we planned an ad-hoc sample size recalculation to calculate the expected power of the study based on updated parameters. The power will be re-calculated using the actual clinical failure data from the control arm, adjusting the upper limit of the non-inferiority margin based on the risk ratio of 1.3, and taking into the actual enrollment numbers extrapolated up to mid-October 2022 (which corresponds to the end of 6 months of enrollment in all health facilities). All the other sample size calculation parameters will remain the same (see above)). The following approaches will therefore be undertaken based on the calculated power:

- If the updated sample size calculation finds a power of ≥80%, than the study will end as planned mid-October 2022.
- If the updated sample size calculation finds a power of <80% we will prolong the study, or increase the number of health facilities as required.

The sample size recalculation will be performed by an independent statistician, who will share the results of the updated sample size to the study investigators. To avoid implementation bias, the clinical failure proportion in the control arm will not be shared.

3.5 Timing of final analysis

The final analysis will be performed after all subjects have the day 7 outcome measured.

4. Statistical principles

4.1 Adherence and protocol deviations

Adherence to the study protocol is assessed based on the percent of patients enrolled in the study for which the clinician entered the final treatment.

Adherence is defined as: % adherence to the study protocol = ((number of children for whom the final treatment were inputted) / (number of children enrolled in the study))*100%

Descriptive statistics on the adherence will be provided by study arm (intervention and control)

4.2 Analysis population definitions

Intention-to-treat population: This population includes all patients enrolled in the study, regardless of whether the health care worker used ePOCT+ (intervention arm), or entered the final treatment data in the eCRF (both arms).

Per-protocol population: This population only includes patients enrolled in the study for whom the clinician confirmed the final treatment (or absence of treatment) / referral status within the ePOCT+ tool (intervention arm) or in the eCRF (control arm).

Complete case set: All enrolled subjects for whom the primary endpoint of day 7 clinical cure data was collected (i.e. excluding lost to follow-up patients).

Complete consultation set: All enrolled subjects (intervention or control) for whom at least information on treatments were collected at day 0.

5. Study population

5.1 Screening data

All patients presenting to the outpatient department of participating health facilities will be screened for eligibility. At the screening stage, the age of the patient and reason for coming to the health facility (acute vs. routine or chronic care visit) will be documented. The following summary data for the facility will be presented: the number of days during which screening has taken place, the number of patients recruited, the average number of patients recruited per day, the number of screened patients not recruited, and the reason for non-recruitment (e.g. non-eligibility vs. declined enrollment).

5.2 Eligibility

5.2.1 Health facility (cluster) eligibility

Heath facility eligibility for the study is based on the health facility meeting all inclusion criteria and none of the exclusion criteria:

Inclusion Criteria:

- Government and private government-designated Primary care HFs (health posts, dispensaries and health centers).

- Located in the Mlimba, Ifakara and Ulanga districts (Morogoro Region) or in Mbeya Rural district and Mbeya city council (Mbeya Region).
- Seeing at least 20 children aged 2 months to 5 years per week on average over the past 6 months (to facilitate participant recruitment)*.

*Based on estimates from extrapolated data from HMIS (MoHCDGEC-HMIS Unit)

Exclusion Criteria:
- Secondary and Tertiary HFs (district, regional, zonal and specialized hospitals).
- Located more than 150 km from the research institution (for feasibility considerations)

5.2.2 Study population eligibility
A patient is eligible for the study if they meet all inclusion criteria and none of the exclusion criteria:

Inclusion Criteria:
- Aged 1 day (24 hours) to 14 years (inclusive)
- Presenting for an acute medical or surgical condition

Exclusion Criteria:
- Presenting for scheduled consultation for a chronic disease (e.g. HIV, TB, NCD, malnutrition)
- Presenting for routine preventive care (e.g. growth monitoring, vitamin supplementation, deworming, vaccination).
- Caregiver unavailable, unable or unwilling to provide informed consent (except for older children who can provide assent with an adult witness during the consenting process).

5.3 Recruitment
A CONSORT flow diagram will be used to summarize the number of patients:

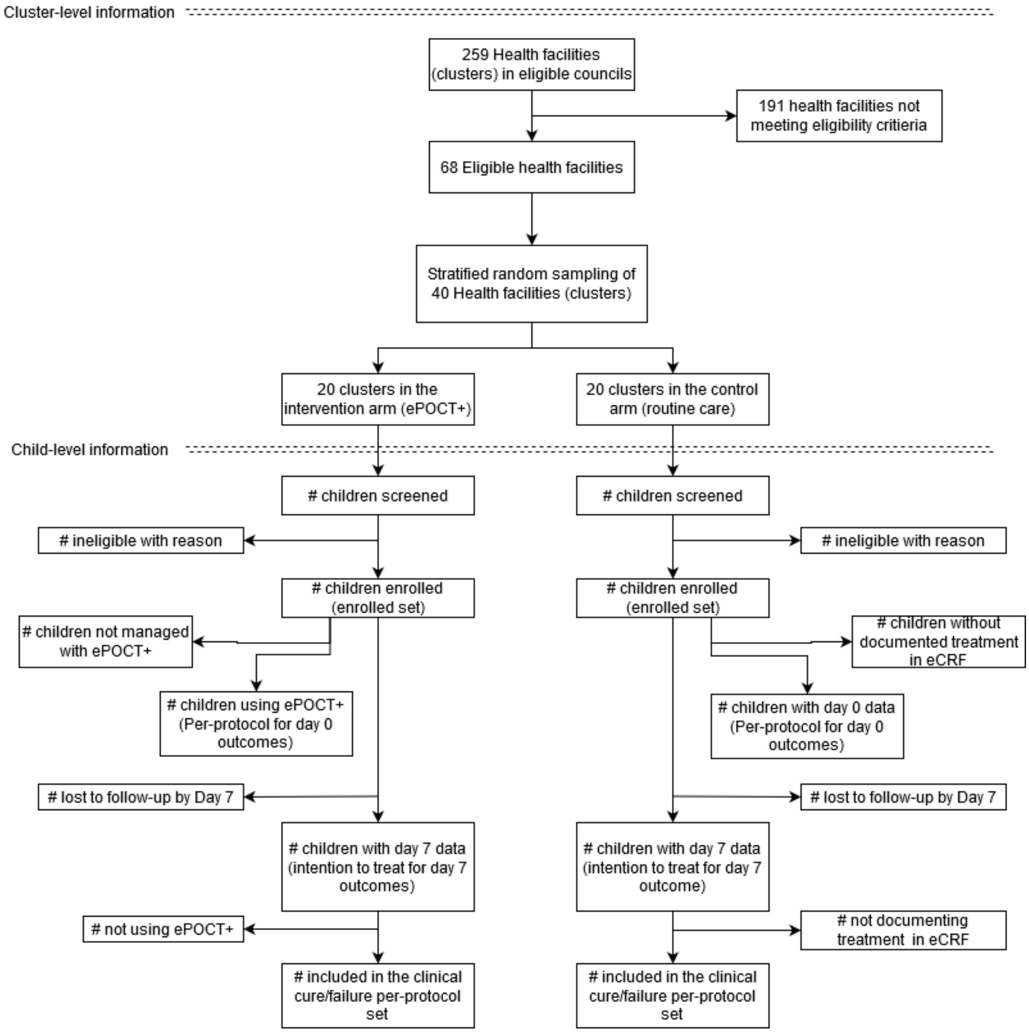

5.4 Baseline characteristics

The baseline cluster (health facility) characteristics that will be summarized by study arm include:

- Number of health facilities by type (dispensary or health center) and region
- Average number of patients seen per month by health facility type
- Availability and readiness of basic health care services score based on the Tanzanian Service Provision Assessment (median and IQR)

The baseline patient characteristics that will be summarized by study arm, and for the overall study include:

- Demographics: sex (number and percentage); age (median and IQR), age groups 0-2 months, 2 months - 5 years, 5-14 years (number and percentage)
- Medical history: Main reasons for consultation, history of fever (number and percentage)

Given the pragmatic nature of the study, and so as not to influence the normal management of patients and their outcome in the control arm, clinical signs and comorbidities will not be systematically collected in the control and intervention arm.

No formal statistical comparisons of baseline data will be performed.

6. Main analyses

6.1 Outcome definitions

6.1.1 Co-primary outcomes

1. Percentage of children cured at day 7 in the intervention group (ePOCT+) as compared to the control group (routine care)

Outcome measure: The child is defined as being cured at day 7 if the caregiver says that the child is cured or has improved since the initial consultation. Non-referred secondary hospitalizations (if caregiver says that child was hospitalized between day 0 and day 7 but the electronic clinical data does not indicate a referral for hospitalization) will however be considered as clinical failures even if the child is already cured at day 7.

Timing and method of measurement: Assessment of subjective clinical cure by the caregiver, and history of non-referred hospitalization will be assessed by telephone or home visit follow-up 7 days (range 6-14 days) after enrollment of the subject. The day of enrollment of the subject is considered as day 0.

Analysis type: Non-inferiority.

2. Percentage of children prescribed an antibiotic at initial consultation in the intervention group (ePOCT+) as compared to the control group (routine care)

Outcome measure: Prescription of oral or parenteral antibiotic at initial consultation, as reported by the health care worker.

Timing and method of measurement: Documented by the health care worker at the end of the initial consultation where the subject was enrolled (day 0).

Analysis type: Superiority

6.1.2 Secondary outcomes

All secondary outcomes are compared between the intervention (ePOCT+) and control (routine care) arms.

1) *Secondary consultations*
- % of children with one or more unscheduled re-attendance visits at any health facility by day 7

Timing and method of measurement: Telephone or home visit follow-up 7 days (range 6-14 days) after enrollment of the subject. The day of enrollment of the subject is considered as day 0.

2) *Severe outcome by day 7*
- % of children with non-referred secondary hospitalization by day 7
- % of children who have died by day 7

Timing and method of measurement: Death and non-referred secondary hospitalization will be assessed by telephone or home visit follow-up 7 days (range 6-14 days) after enrollment of the subject. The day of enrollment of the subject is considered as day 0.

3) *Primary referrals*
- % of children referred to hospital or inpatient ward at a health centre at initial consultation

Timing and method of measurement: Documented by the health care worker at the end of the initial consultation in the eCRF (control arm) or in ePOCT+ (intervention arm) when the subject was enrolled (day 0)

4) *Appropriate case management for malaria at initial consultation*

- % of febrile children tested for malaria by RDT and/or microscopy at day 0
- % of malaria positive children (positive RDT result OR positive microscopy result) prescribed an antimalarial at day 0
- % of malaria negative children prescribed an antimalarial at day 0
- % of untested children prescribed an antimalarial at day 0

Timing and method of measurement: Documented by the health care worker at the end of the initial consultation where the subject was enrolled (day 0)

Definitions:

Clinical cure is a positive answer to the question "Is the child cured?" or if not cured "Has the child improved?" asked to caregiver through a phone call at day 7. Non-referred secondary hospitalizations (see definition below) will be considered as clinical failures even if the child is already cured at day 7.
Clinical failure is a negative answer to the question "Is the child cured?" and "Has the child improved" asked to the caregiver through a phone call at day 7. Non-referred secondary hospitalization (see definition below) will be considered as a clinical failure even if the child is already cured at day 7.
Initial consultation is the first visit of a sick child for an acute problem at a HF participating in the study (and thus registered electronically); timeframe from completion of the initial visit up to midnight of the same day. The initial consultation is considered as day 0.
Re-attendance visit is a consultation (not necessarily at a HF participating in the study) taking place from the day after initial consultation up to day 14 included. A re-attendance visit can be scheduled (proposed by the health care worker on a certain day) or unscheduled (upon decision by caregivers).
Primary referral is a decision of referring or admitting the child at a hospital or health center for at least one night, taken by the health care worker at the initial consultation.
Secondary referral is a decision of referring or admitting the child at a hospital or health center taken by the health care worker during a re-attendance visit taking place from the day after initial consultation up to day 7 included.
Primary hospitalization is an admission to a hospital or health center ward for at least one night taking place the same day as the initial consultation
Secondary hospitalization is an admission to a hospital or health center ward for at least one night taking place from the day after initial consultation up to the phone call at day 7 included. A non-referred secondary hospitalization is a direct visit to hospital without a re-attendance visit the same day at a HF participating in the study. Non-referred secondary hospitalization will be determined if the caregiver reports during the day 7 phone follow-up that the child was hospitalized, but there was no documentation of a referral from initial and re-attendance visits at participating health facilities.
Antibiotic prescription is any oral, intramuscular or intravenous (but not topical) antibiotic prescribed by a HCW during the initial consultation or a re-attendance visit.
Antimalarial prescription is any oral, rectal, intramuscular or intravenous antimalarial prescribed by a HCW during the initial consultation or a re-attendance visit.
Febrile child is a child with a history of fever (measured or suspected fever in the past 48 hours) or a high temperature.
Malaria testing is a malaria RDT or microscopy ordered by a HCW during an outpatient visit.

6.2 Analysis methods

Analyses will follow CONSORT guidelines (Campbell, Piaggio, Elbourne, & Altman, 2012; Piaggio et al., 2012; Schulz, Altman, & Moher, 2010; Zwarenstein et al., 2008). Outcomes will be described by arm using summary statistics. For the non-inferiority comparison between ePOCT+ and the control arm for clinical failure, a CI approach will be used using a random effects logistic regression model using the cluster (health facility) and patient as random effects. A figure illustrating the 95% CIs of the ITT and PP relative risk of clinical failure will be presented with the pre-specified non-inferiority margin of 1.3. Non-inferiority will be achieved if the upper-bound of the RR does not surpass 1.3. If ePOCT+ is found to be

non-inferior to the control in terms of clinical failure using the PP population, then we will assess for superiority using the ITT population. Superiority will be achieved if the relative risk CI of the ITT set does not surpass 1. If clinical failure non-inferiority is established, we will test for significant differences in the co-primary outcome of antibiotic prescription rate. Since the documentation of the final treatment in both arms is part of the per protocol definition, only the PP population can be used. For this outcome, the percentage of patients receiving antibiotics at the initial consultation will also be assessed using a random effects logistic regression model with the cluster (health facility) and patient included as random effects. Binary secondary outcomes and additional analyses will be evaluated in the same way. All estimates will be reported with two-sided 95% confidence intervals (CI), except for the non-inferiority analysis which will be reported with one-sided 95% CI. The study will be deemed a success if clinical failure in the ePOCT+ arm is non-inferior to the control arm, and antibiotic prescription at the initial consultation is 25% less in the ePOCT+ arm compared to the control arm.

Effect modification of the co-primary outcomes of antibiotic prescription and clinical failure will be performed in groups of patients where the highest reduction in antibiotic prescription is expected, and areas of extension of the content of the algorithm compared to the version tested in the previous ePOCT study. Effect modification by sex, age group (young infants ages 1 to 59 days, children aged 2 months to 5 years, and children aged 5 to 14 years), respiratory symptoms, fever without clinical source, gastrointestinal complaints (vomiting and diarrhea), skin problems, ear, nose and throat problems, will be assessed by incorporating an interaction between arm and the respective variable, acknowledging that power will be low. All models will be adjusted for clustering and the stratification factors (Kahan & Morris, 2012). Furthermore in order to adjust for potential imbalances in baseline characteristics between study groups, relevant baseline characteristics will be included as fixed effect variables in the final model. Baseline characteristic imbalance will be determined by consensus among the study analysis team.

The number of children lost to follow up may be quite high due to the difficult nature of phone follow-up at day 7, as found in previous studies with day 7 phone call lost to follow up rates of 13-25%(Christie et al., 2018; Hannigan, Chisale, Drew, Watson, & Gallagher, 2019; Nguhuni et al., 2017). Comparatively, the proportion not cured at day 7 is expected to be quite low (3%). The use of a complete case analysis was therefore selected for the non-inferiority co-primary outcome of day 7 clinical cure, as it would be the most conservative analysis. If we were to use the “enrolled set” and considered all day 7 lost to follow up as not being cured, this would dilute the difference between arms, and thus may demonstrate non-inferiority when there is truly a difference between arms (type 1 error). Not considering lost to follow up cases as failures is also the approach used for similar studies with high number of LTF and low proportion of outcomes (Källander et al., 2018). We may adjust the final model for further baseline variables (not already included as fixed effect variables) which are associated with missing outcome data (analogous to performing multiple imputation in the case of a single endpoint). As with the other fixed effect variables, baseline characteristic imbalances among patients lost to follow up will be determined by consensus among the study analysis team.

6.3 Exploratory analyses

Unless specifically described, the following analyses will be analyzed similarly to the binary secondary outcomes, using a random effects logistic regression model with the cluster (health facility) and patient included as a random effect.

- % of children hospitalized among those referred for hospitalization during an initial consultation

Timing and method of measurement: Hospitalization determined by day 7 (6-14) phone call or home visit. Referral for hospitalization as determined by eCRF during the initial consultation.

- % of children referred for malnutrition management during the initial consultation

Timing and method of measurement: Referred for malnutrition as documented in eCRF during the initial consultation.

- % of children referred for or performed at the health facility tuberculosis screening during the initial consultation

Timing and method of measurement: As documented in the eCRF during the initial consultation.

- % of children tested for HIV during the initial consultation:

Timing and method of measurement: As documented in the eCRF during the initial consultation.

- % of children tested for any laboratory test during the initial consultation:

Timing and method of measurement: As documented in the eCRF during the initial consultation

- % of clinicians accepting the ePOCT+ testing, treatment, diagnosis, and management recommendations during the initial consultation

Timing and method of measurement: As documented in the eCRF of the intervention arm (ePOCT+) during the initial consultation

- % of children/caretakers seeking additional medications after the initial consultation

Timing and method of measurement: Asking caretakers if they procured additional medications for their child after the initial consultation by phone call or home visit at day 7 (6-14)

- Change in % of children with antibiotic prescribed over time

Timing and method of measurement: Antibiotic prescription as documented in the eCRF (control arm) / ePOCT+ tool (intervention) during the initial visit (day 0).

Analysis: Compare change in percentage of prescription from month to month, and from first half of the study compared to second half of the study for the intervention arm and the control arm, using the control arm as comparison to correct for seasonality, and presenting implementation team mentorship visits and combined implementation team and CHMT mentorship visits to contextualize changes. Independent sample t-test will be used to compare change in antibiotic prescription between intervention and control arm.

- Change in % of children with basic anthropometrics and clinical signs assessed (intervention arm only)

Timing and method of measurement: Measurement and documentation of individual basic measurements (height, MUAC, Temperature), and clinical signs (respiratory rate) as documented within the ePOCt+ tool during the initial assessment (Day 0).

Analysis: Descriptive statistics presenting change in documentation of basic measurements and clinical signs from month to month, and from first half of the study compared to second half of the study. Dates of implementation team mentorship visits and combined/supervised implementation team and CHMT mentorship visits and any changes in implementation strategy will also be presented to contextualize the changes. Subgroup analyses may be conducted for each intervention health facility.

- Change in % of cases managed using ePOCT+ over time (intervention arm only):

Timing and method of measurement: Uptake of ePOCT+ is defined as the use of the ePOCT+ tool to guide the clinical consultation including the decision on what treatment to prescribe during the initial consultation (Day 0).

Analysis: Descriptive statistics to present change in the proportion of health care workers' uptake from month to month, and from first half of the study compared to second half of the study will be presented. Dates of implementation team mentorship visits and combined/supervised implementation team and CHMT mentorship visits and any changes in implementation strategy will also be presented to contextualize the changes. Subgroup analyses may be conducted for each intervention health facility.

- Prognostic value of individual or combined predictors during the initial consultation to predict clinical failure/cure, hospitalization for more than one night and mortality (intervention arm)

Timing and method of measurement: Individual or combined predictors as documented in the eCRF during the initial consultation in the intervention arm. Clinical failure, hospitalization more than one night, and mortality assessed during the phone calls or home visit at day 7.

Method of analysis: ORs and 95% confidence intervals will be calculated using Pearson's chi-squared test or Fisher's exact test (two-tailed) for binary individual predictors. Initial selection of individual

predictors for the bivariate models will be determined by a group of clinical experts, with consideration of the algorithmic logic of the intervention tool to avoid circularity, and evidence on predictors already found to be prognostic. Multivariable ordinal logistic regression with lasso penalty will be used for covariate selection of the final model. Validity tests will be calculated including sensitivity, specificity, positive likelihood ratio, negative likelihood ratio, and area under the receiver operating characteristic. The dataset will be divided into a portion used for the derivation, and another portion for validation.

References

Campbell, M. K., Piaggio, G., Elbourne, D. R., & Altman, D. G. (2012). Consort 2010 statement: extension to cluster randomised trials. *BMJ : British Medical Journal, 345*, e5661. doi:10.1136/bmj.e5661

Christie, S. A., Mbianyor, M. A., Chichom-Mefire, A., Nana, T., Dicker, R. A., & Juillard, C. (2018). Feasibility of a Cellular Telephone Follow-Up Program after Injury in Sub-Saharan Africa. *Journal of the American College of Surgeons, 227*(4), S129-S130. doi:10.1016/j.jamcollsurg.2018.07.272

Hannigan, A., Chisale, M., Drew, R., Watson, C., & Gallagher, J. (2019). GP133 Mobile phones for follow up in paediatric clinical studies in africa. *104*(Suppl 3), A84-A84. doi:10.1136/archdischild-2019-epa.197 %J Archives of Disease in Childhood

Kahan, B. C., & Morris, T. P. (2012). Reporting and analysis of trials using stratified randomisation in leading medical journals: review and reanalysis. *BMJ (Clinical research ed.), 345*, e5840-e5840. doi:10.1136/bmj.e5840

Källander, K., Alfvén, T., Funk, T., Abebe, A., Hailemariam, A., Getachew, D., . . . Gutman, J. R. (2018). Universal versus conditional day 3 follow-up for children with non-severe unclassified fever at the community level in Ethiopia: A cluster-randomised non-inferiority trial. *PLoS Med, 15*(4), e1002553-e1002553. doi:10.1371/journal.pmed.1002553

MoHCDGEC-HMIS Unit. 2019. Retrieved from http://dhis.moh.go.tz/dhis-web-pivot/app/index.html]

Nguhuni, B., De Nardo, P., Gentilotti, E., Chaula, Z., Damian, C., Mencarini, P., . . . Ippolito, G. (2017). Reliability and validity of using telephone calls for post-discharge surveillance of surgical site infection following caesarean section at a tertiary hospital in Tanzania. *Antimicrobial Resistance & Infection Control, 6*(1), 43. doi:10.1186/s13756-017-0205-0

Piaggio, G., Elbourne, D. R., Pocock, S. J., Evans, S. J. W., Altman, D. G., & Consort Group, f. t. (2012). Reporting of Noninferiority and Equivalence Randomized Trials: Extension of the CONSORT 2010 Statement. *JAMA, 308*(24), 2594-2604. doi:10.1001/jama.2012.87802

Schulz, K. F., Altman, D. G., & Moher, D. (2010). CONSORT 2010 Statement: updated guidelines for reporting parallel group randomised trials. *BMJ, 340*, c332. doi:10.1136/bmj.c332

Zwarenstein, M., Treweek, S., Gagnier, J. J., Altman, D. G., Tunis, S., Haynes, B., . . . Moher, D. (2008). Improving the reporting of pragmatic trials: an extension of the CONSORT statement. *BMJ, 337*, a2390. doi:10.1136/bmj.a2390
