# Supplementary material for: A digital health algorithm to guide antibiotic prescription in pediatric outpatient care: a cluster randomized controlled trial
Source: Nat Med. 2023 Dec 18;30(1):76–84. doi: 10.1038/s41591-023-02633-9 (PMC10803249; doi:10.1038/s41591-023-02633-9)
Supplement: Supplementary file 2 — Reporting Summary [file 41591_2023_2633_MOESM2_ESM.pdf]

nature portfolio

Corresponding author(s): Rainer Tan

Last updated by author(s): 9/28/2023

Reporting Summary

Nature Portfolio wishes to improve the reproducibility of the work that we publish. This form provides structure for consistency and transparency in reporting. For further information on Nature Portfolio policies, see our Editorial Policies and the Editorial Policy Checklist.

Statistics

For all statistical analyses, confirm that the following items are present in the figure legend, table legend, main text, or Methods section.

n/a	Confirmed	
☐	☒	The exact sample size (*n*) for each experimental group/condition, given as a discrete number and unit of measurement
☐	☒	A statement on whether measurements were taken from distinct samples or whether the same sample was measured repeatedly
☐	☒	The statistical test(s) used AND whether they are one- or two-sided<br>*Only common tests should be described solely by name; describe more complex techniques in the Methods section.*
☐	☒	A description of all covariates tested
☐	☒	A description of any assumptions or corrections, such as tests of normality and adjustment for multiple comparisons
☐	☒	A full description of the statistical parameters including central tendency (e.g. means) or other basic estimates (e.g. regression coefficient) AND variation (e.g. standard deviation) or associated estimates of uncertainty (e.g. confidence intervals)
☐	☒	For null hypothesis testing, the test statistic (e.g. *F*, *t*, *r*) with confidence intervals, effect sizes, degrees of freedom and *P* value noted<br>*Give P values as exact values whenever suitable.*
☒	☐	For Bayesian analysis, information on the choice of priors and Markov chain Monte Carlo settings
☐	☒	For hierarchical and complex designs, identification of the appropriate level for tests and full reporting of outcomes
☒	☐	Estimates of effect sizes (e.g. Cohen's *d*, Pearson's *r*), indicating how they were calculated

Our web collection on statistics for biologists contains articles on many of the points above.

Software and code

Policy information about availability of computer code

Data collection	The code for the medAL-reader application used to collect data entered by health providers (including demographic, clinical, diagnosis, prescription, and referral data of the consultations) can be found here: https://github.com/Wavemind/liwi-medal-reader<br><br>A separate publication describing medAL-reader and the ePOCT+ algorithm can be found here: https://journals.plos.org/digitalhealth/article?id=10.1371/journal.pdig.0000170<br><br>Day 7 outcome on clinical cure, additional health facility visits, hospitalization, death and additional medicines taken were collected using RedCAP versions 11.2.2 to 12.5.9: https://www.project-redcap.org/
Data analysis	All data analysis was performed using STATA v16 and v17

For manuscripts utilizing custom algorithms or software that are central to the research but not yet described in published literature, software must be made available to editors and reviewers. We strongly encourage code deposition in a community repository (e.g. GitHub). See the Nature Portfolio guidelines for submitting code & software for further information.

Data

Policy information about availability of data

All manuscripts must include a data availability statement. This statement should provide the following information, where applicable:

- Accession codes, unique identifiers, or web links for publicly available datasets
- A description of any restrictions on data availability
- For clinical datasets or third party data, please ensure that the statement adheres to our policy

De-identified data can be found on https://doi.org/10.5281/zenodo.8043523, including case, patient and health facility identification number, study arm allocation, baseline characteristics, and all outcomes.

Research involving human participants, their data, or biological material

Policy information about studies with human participants or human data. See also policy information about sex, gender (identity/presentation), and sexual orientation and race, ethnicity and racism.

Reporting on sex and gender	Only sex was reported in this study as suggested by the Tanzanian research institutes involved in the study. Sex specific subgroup analyses were pre-specified, performed, and shared for the co-primary outcomes.
Reporting on race, ethnicity, or other socially relevant groupings	Race, ethnicity, or other socially relevant groupings was not collected in this study.
Population characteristics	Infants and children aged between 1 day old and under 15 years of age seeking care for an acute medical or surgical condition at participating health facilities were included. 51% of patients were female, 5% were 0-2 months, 83% were 2-59 months, and 12% were 5-14 years. 91% of consultations were new/initial consultations, and 9% re-attendance consultations.
Recruitment	Children seeking care at included health facilities were screened for eligibility by a research assistant between 8:00 to 16:00 on weekdays. Patients presenting to care outside of normal working hours would not have been screened or enrolled which may bias representation of participants. While not an exclusion criteria, illiterate caregivers had to find a witness to sign the consent forms, this additional barrier may have reduced the number of participants for which their caregiver was illiterate.
Ethics oversight	Ethical approval was obtained in Tanzania from the Ifakara Health Institute (IHI/IRB/No: 11-2020), the Mbeya Medical Research Ethics Committee (SZEC-2439/R.A/V.1/65), the National Institute for Medical Research Ethics Committee (NIMR/HQ/R.8a/Vol. IX/3486 and NIMR/HQ/R.8a/Vol. IX/3583), and in Switzerland from the cantonal ethics review board of Vaud (CER-VD 2020-02800).

Note that full information on the approval of the study protocol must also be provided in the manuscript.

Field-specific reporting

Please select the one below that is the best fit for your research. If you are not sure, read the appropriate sections before making your selection.

☒ Life sciences ☐ Behavioural & social sciences ☐ Ecological, evolutionary & environmental sciences

For a reference copy of the document with all sections, see nature.com/documents/nr-reporting-summary-flat.pdf

Life sciences study design

All studies must disclose on these points even when the disclosure is negative.

Sample size	The sample size was calculated for testing non-inferiority of the clinical failure outcome given that it would require a higher sample size than for the antibiotic prescription co-primary outcome. We assumed a cluster size of 900 patients (average of 150 patients per month x 6 months) based on routine data within the natinoal health management information system, an intraclass correlation coefficient of 0.002, and a clinical failure rate of 3%. To have 80% power to detect an acceptable non-inferiority margin of a relative risk of 1.3, corresponding to 3.9%, we required 19 clusters and 17,100 patients per arm (total patients n=37,620 assuming 10% loss to follow-up). Given the uncertainty of some of the assumptions, the total number of health facilities was rounded up to 20 clusters per arm.
Data exclusions	or the primary analysis of antibiotic prescription data exclusion include: patients consulting for a re-attendance visit (consulting a health facility following an initial consultation for the same acute illness), patients not managed using the ePOCT+ tool for the whole consultation (not per-protocol)<br>For the primary analysis of clinical failure, data exclusion include: patients consulting for a re-attendance visit (consulting a health facility following an initial consultation for the same acute illness), patients not managed using the ePOCT+ tool for the whole consultation (not per-protocol), and patients for which day 7 outcome was not ascertained (not complete case)
Replication	Not applicable
Randomization	The sampled health facilities were randomized (1:1), to ePOCT+ (intervention) or usual care (control). Randomization was stratified by region,

Randomization	level of health facility (health center versus dispensary), attendance rate, and council. An independent statistician in Switzerland was provided the list of all eligible health facilities, and performed the computer-generated sampling and randomization. Intervention allocation was only shared to study investigators in Tanzania once all council leaders confirmed the participation of their selected health facilities.
Blinding	The nature of the intervention did not allow for masking of the intervention to healthcare providers, patients, or study implementers.

Reporting for specific materials, systems and methods

We require information from authors about some types of materials, experimental systems and methods used in many studies. Here, indicate whether each material, system or method listed is relevant to your study. If you are not sure if a list item applies to your research, read the appropriate section before selecting a response.

Materials & experimental systems

n/a	Involved in the study
☒	☐ Antibodies
☒	☐ Eukaryotic cell lines
☒	☐ Palaeontology and archaeology
☒	☐ Animals and other organisms
☐	☒ Clinical data
☒	☐ Dual use research of concern
☒	☐ Plants

Methods

n/a	Involved in the study
☒	☐ ChIP-seq
☒	☐ Flow cytometry
☒	☐ MRI-based neuroimaging

Clinical data

Policy information about clinical studies

All manuscripts should comply with the ICMJE guidelines for publication of clinical research and a completed CONSORT checklist must be included with all submissions.

Clinical trial registration	ClinicalTrials.gov number NCT05144763
Study protocol	Found on the ClinicalTrials.gov website number NCT05144763
Data collection	Consultation data was collected between 1 December 2021, and 31 October 2022 by research assistants and healthcare workers in the eligible health facilities on weekdays between 8:00 to 16:00. Day 7 outcome data was collected by research assistants by phone or home visit on weekdays between 08:00 to 16:00.
Outcomes	The co-primary outcomes measured at the individual patient level included: 1) antibiotic prescription at the time of the initial consultation as documented by the healthcare provider (superiority analysis); and 2) clinical failure at day 7 defined as “not cured” and “not improved”, or unscheduled hospitalization as reported by caregivers (non-inferiority analysis). Secondary outcomes include unscheduled re-attedance visits at any health facility by day 7, non-referred secondary hospitalization by day 7, death by day 7, and referral for inpatient hospitalization at initial consultation.
